# Epidemiological and Evolutionary Dynamics of Dengue Virus in Saudi Arabia: Insights from Three Decades of Molecular and Serological Surveillance

**DOI:** 10.3390/ijms27136014

**Published:** 2026-07-04

**Authors:** Mohamed A. Farrag, Reem M. Aljowaie, Ibrahim M. Aziz, Rawan M. Alshalan, Abdulaziz Abdullah Almosa, Basel Mohammed Alnafjan, Najat A. Y. Marraiki

**Affiliations:** 1 Department of Botany and Microbiology, College of Science, King Saud University, Riyadh 11451, Saudi Arabia; raljowaie@ksu.edu.sa (R.M.A.); iaziz@ksu.edu.sa (I.M.A.); ralshalaan@ksu.edu.sa (R.M.A.); najat@ksu.edu.sa (N.A.Y.M.); 2 Wellness Prevention Medicine, King Abdulaziz City for Science and Technology (KACST), Riyadh 11451, Saudi Arabia; azimousa@kacst.gov.sa (A.A.A.); bnafjan@kacst.gov.sa (B.M.A.)

**Keywords:** dengue virus, Saudi Arabia, envelope gene, phylogenetic analysis, serotypes, molecular epidemiology, glycosylation, DENV-2, cosmopolitan genotype, genomic surveillance

## Abstract

Dengue fever represents a significant public health challenge in Saudi Arabia, yet comprehensive molecular characterization of circulating serotypes remains limited. This study combines epidemiological and phylogenetic analyses to understand dengue virus (DENV) dynamics in the Kingdom. A systematic review and meta-analysis of dengue epidemiological data from Saudi Arabia (1992–2026) was the Preferred Reporting Items for Systematic Reviews and Meta-Analyses (PRISMA) guidelines. All available DENV envelope (*E*) gene sequences from Saudi human cases (1992–2023) were retrieved from GenBank and Global Initiative on Sharing All Influenza Data (GISAID). Phylogenetic trees were constructed using maximum likelihood with 1000 bootstrap replicates and best-fit models. Selection pressure was analyzed using SLAC, FEL, FUBAR, and MEME methods, while glycosylation sites were predicted with NetNGlyc and NetOGlyc. The pooled seroprevalence from 25 studies (*n* = 32,393) was 40.71% (95% CI: 26.96–56.10%). DENV-2 predominated (80.25%), followed by DENV-1 and DENV-3, with DENV-4 remaining rare (0.42%). Males (67–78%) and adults aged 25–44 years were most affected. Phylogenetic analysis of 50 Saudi isolates showed DENV-1 strains clustered within American–African (1994) and Asian (2004–2011) genotypes, all DENV-2 within the Cosmopolitan genotype, and all DENV-3 within Genotype III (bootstrap support 99–100%). Selection pressure analysis indicated pervasive positive selection in DENV-2, episodic selection across serotypes, and strong purifying selection in the *E* gene. Several amino acid substitutions with potential functional importance were identified. No DENV-4 *E* gene sequences from Saudi Arabia are publicly available. Dengue in western Saudi Arabia is characterized by DENV-2 predominance, co-circulation of three serotypes, and multiple introductions. The absence of DENV-4 sequences highlights critical surveillance gaps. Sustained molecular surveillance, expanded genomic sequencing, and data sharing are essential for effective prevention and vaccine preparedness.

## 1. Introduction

Dengue fever (DF) is the most significant arthropod-borne viral disease worldwide and poses a substantial public health burden in tropical and subtropical regions [1,2]. The causative agent, dengue virus, belongs to the genus Flavivirus within the family Flaviviridae and exists as four distinct serotypes (DENV-1, DENV-2, DENV-3, and DENV-4) [3,4]. DENV is transmitted primarily by *Aedes aegypti* and, to a lesser extent, *Aedes albopictus* mosquitoes, which thrive in urban and semiurban environments [5]. Infection with any serotype can produce a spectrum of clinical manifestations ranging from asymptomatic or mild febrile illness to severe dengue hemorrhagic fever (DHF) and dengue shock syndrome (DSS), the latter of which can be fatal without prompt supportive care [6]. The global incidence of dengue has increased dramatically over the past five decades, with an estimated 96 million symptomatic cases annually prior to the COVID-19 pandemic [7]. In 2023 alone, over 6.5 million dengue cases and approximately 6800 deaths were reported globally, whereas in 2024, a new historical record with more than 14 million cases and more than 12,000 deaths was established, and high transmission rates are expected to persist into 2025 [8]. Endemicity has expanded beyond the traditionally affected regions of Southeast Asia and Latin America to include the Middle East. Saudi Arabia now bears one of the highest dengue burdens in the region [9,10].

The first documented case of dengue in Saudi Arabia was reported in Jeddah in late 1993, followed by 289 confirmed cases in 1994 [11]. After a decade of sporadic outbreaks involving fewer than 15 cases annually, the period between 2004 and 2015 witnessed substantially larger epidemics that extended beyond Jeddah to Makkah, Al-Madinah, Jizan, and Najran [11,12]. Consequently, the western region of Saudi Arabia was declared dengue-endemic by the Ministry of Health in 2004, a designation that persists today [9,13,14]. A recent systematic review and meta-analysis covering the period 2003–2023 estimated that the pooled seroprevalence of dengue in Saudi Arabia was 30.8%, with the highest rates observed in the western provinces [15]. Epidemiological surveillance data indicate that DENV-2 has historically been the predominant serotype, followed by DENV-1 and DENV-3, whereas DENV-4 remained undetected until a report in asymptomatic blood donors from the western region [14,16,17,18]. A recent hospital-based study from Makkah (April 2023–May 2024) confirmed the continued predominance of DENV-2, followed by DENV-1 and DENV-3, and a low prevalence of DENV-4 [18].

The unique epidemiological landscape of dengue in Saudi Arabia is shaped by several distinctive factors [13]. The country hosts approximately 32 million residents, nearly one-third of whom are expatriate workers, primarily from dengue-endemic countries in South Asia and Southeast Asia [19]. Additionally, Saudi Arabia receives over 5 million religious pilgrims annually for the Hajj and Umrah pilgrimages, with visitors arriving from more than 180 countries, many of which are endemic for dengue [20,21]. Phylogenetic studies have demonstrated multiple introductions of DENV strains into Saudi Arabia, likely through these population movements, with isolates showing close genetic relationships to strains circulating in Indonesia, Pakistan, India, and Somalia [18,20]. A recent whole-genome phylogeographic analysis of strains collected from 2021 to 2023 confirmed repeated introductions from South Asia and Africa, with DENV-2 remaining the dominant serotype and evidence of cryptic local transmission [9,17].

Despite the established endemicity and public health significance of dengue in Saudi Arabia, comprehensive molecular characterization of circulating DENV serotypes via publicly available genomic data remains limited. The *E* protein-encoding gene is a particularly valuable phylogenetic marker because of its role in viral attachment, membrane fusion, and neutralization, as well as its involvement in serotype-specific immune responses [22,23]. Analysis of *E* gene sequences enables the identification of circulating genotypes, tracking of viral movement across geographical boundaries, and detection of amino acid substitutions that may influence viral fitness, virulence, or antigenicity. Recent *E* gene analyses from neighboring endemic regions, such as Pakistan and India, have demonstrated the utility of this approach in identifying clade replacements and potential antiviral targets [22,23,24].

To date, several molecular epidemiological studies have examined DENV strains from Saudi Arabia, with a primary focus on the Jeddah, Makkah, and Jizan regions [17,18,25,26,27]. However, these investigations have been geographically limited, temporally restricted, or have relied on partial genomic regions. Notably, although DENV-4 has been detected at low prevalence by serology and PCR in Makkah [16,18], no *E* gene sequences from Saudi human cases are currently available in GenBank or GISAID. This gap warrants further investigation, as it may reflect true low circulation, limited sequencing efforts, or delays in data sharing.

The present study aims to address these knowledge gaps through an integrative analysis combining systematic review, meta-analysis, and in silico molecular evolution of all available DENV *E* gene sequences from Saudi Arabia deposited in GenBank and GISAID. Specifically, we aimed to (i) conduct a systematic review and meta-analysis to estimate the pooled seroprevalence of dengue in Saudi Arabia and examine heterogeneity by geographic region, diagnostic assay, time period, and population type; (ii) perform phylogenetic analysis using maximum likelihood with best-fit nucleotide substitution models to elucidate the genetic relationships between Saudi Arabian strains and those from other endemic regions, and to determine circulating genotypes; (iii) identify amino acid variations in the *E* protein, including serotype-specific, genotype-specific, and Saudi-unique substitutions, and predict their potential functional implications; and (iv) apply complementary selection pressure analyses (SLAC, FEL, FUBAR, and MEME) to detect signatures of pervasive and episodic positive selection across the DENV *E* gene. This comprehensive analysis will provide critical insights into the molecular epidemiology and evolutionary dynamics of dengue in Saudi Arabia, inform surveillance strategies, and support evidence-based public health interventions.

## 2. Results

### 2.1. Study Selection and Characteristics

**Figure 1:**
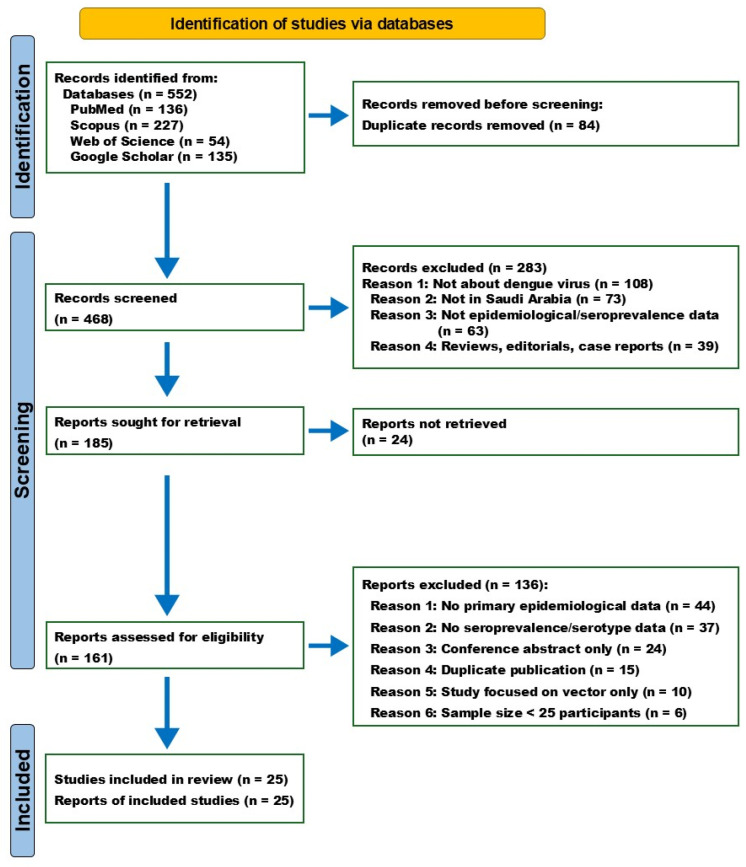
PRISMA flow diagram of the study selection process for the systematic review and meta-analysis of dengue epidemiology in Saudi Arabia (1992–2026).

A systematic literature search across PubMed, Scopus, Web of Science, and Google Scholar identified 552 records. After removing duplicates (*n* = 84), 468 records were screened based on titles and abstracts. Of these, 283 records were excluded as they did not meet the inclusion criteria. The remaining 185 reports were sought for full-text retrieval, of which 24 could not be accessed. The full text of 161 articles was assessed for eligibility, and 136 articles were excluded for various reasons, including lack of primary epidemiological data (*n* = 44), absence of seroprevalence or serotype data (*n* = 37), conference abstracts only (*n* = 24), duplicate publications (*n* = 15), vector-only studies (*n* = 10), and sample size of less than 25 participants (*n* = 6). Ultimately, 25 studies met the inclusion criteria and were included in the systematic review and meta-analysis (Figure 1, PRISMA Flow Diagram).

The 25 included studies were published between 2006 and 2026, with sample sizes ranging from 25 to 6637 participants (total *n* = 32,393). The studies were conducted across multiple regions of Saudi Arabia, including Jeddah (*n* = 6), Makkah (*n* = 6), Jazan (*n* = 5), multiregional (*n* = 3), Al-Madinah (*n* = 2), Aseer/Jazan (*n* = 2), and Najran (*n* = 1). The majority of studies were cross-sectional in design (*n* = 22), with one case–control study, one retrospective study, and one prospective study. Diagnostic assays employed included ELISA, RT-PCR, NS1 antigen detection, and serology. A summary of the characteristics of the included studies is presented in Table 1. Quality assessment using the Joanna Briggs Institute (JBI) Critical Appraisal Tool showed that 18 studies (72%) were at low risk of bias, while 7 studies (28%) were at moderate risk of bias. No studies were classified as high risk of bias.

### 2.2. Meta-Analysis and Subgroup Analyses

A random-effects meta-analysis of 25 studies (*n* = 32,393 participants) yielded a pooled dengue seroprevalence of 40.71% (95% CI: 26.96–56.10%). Individual study estimates ranged widely from 0.83% to 100% (Table S1). The analysis revealed substantial heterogeneity, with an I^2^ statistic of 98.9% (95% CI: 98.7–99.0%) and a τ^2^ value of 2.3906 (τ = 1.5462). The Cochran’s Q test was significant (Q = 2096.80, df = 24, *p* < 0.001), indicating considerable variation across studies (Figure S1, Forest Plot).

Stratification by geographic region revealed the highest pooled seroprevalence in Makkah at 76.16% (95% CI: 17.51–97.96%), followed by Jazan at 54.77% (95% CI: 46.20–63.06%) and Jeddah at 40.09% (95% CI: 30.87–50.07%). Lower estimates were observed in multiregional studies at 36.65% (95% CI: 24.08–51.34%), Al-Madinah at 34.30% (95% CI: 7.24–77.74%), Najran at 7.56% (95% CI: 5.37–10.55%), and Aseer/Jazan at 5.95% (95% CI: 0.12–76.42%). The between-group difference was statistically significant (Q = 122.39, df = 6, *p* < 0.0001) (Table S2).

When stratified by diagnostic assay (Table S3), RT-PCR demonstrated the highest pooled prevalence at 60.52% (95% CI: 54.68–66.07%), followed by PCR/NS1/IgM at 57.92% (95% CI: 51.00–64.54%) and IgM/IgG/PCR at 53.11% (95% CI: 47.50–58.65%). Lower estimates were obtained using IgM alone at 0.83% (95% CI: 0.42–1.65%), IgG/IgM at 25.05% (95% CI: 14.48–39.74%), and IgG at 25.33% (95% CI: 11.23–47.63%). The between-group difference was statistically significant (Q = 383.24, df = 14, *p* < 0.0001).

Temporal analysis showed the highest seroprevalence in studies conducted between 2003 and 2010 at 84.55% (95% CI: 14.46–99.44%), followed by 2019–2026 at 45.73% (95% CI: 26.76–66.02%) and 2011–2018 at 27.11% (95% CI: 14.00–45.93%); however, the between-group difference was not statistically significant (Q = 3.48, df = 2, *p* = 0.1760) (Table S4). Stratification by population type revealed the highest seroprevalence among febrile patients at 49.78% (95% CI: 40.84–58.72%), followed by community-based studies at 26.70% (95% CI: 25.64–27.78%) and blood donors at 24.93% (95% CI: 8.00–55.91%) (Table S5). The lowest estimates were observed among pregnant women at 7.56% (95% CI: 5.37–10.55%). The between-group difference was statistically significant (Q = 110.39, df = 5, *p* < 0.0001). Serotype distribution varied across regions. DENV-2 was the most frequently reported serotype, predominating in Jazan, Makkah, and Jeddah. DENV-1 was the second most common, while DENV-3 was detected in Jazan, Makkah, and Jeddah. DENV-4 was rarely detected, reported only in Makkah. Concurrent mixed infections with DENV-1 and DENV-2 were identified in Jazan. In one study from Makkah, 11 samples tested positive for DENV by RT-PCR but were non-reactive to serotypes 1–4.

### 2.3. Publication Bias

Publication bias was assessed using a funnel plot and Egger’s regression test (Table S6). Visual inspection of the funnel plot revealed some asymmetry; however, Egger’s test was not statistically significant (t = −0.80, df = 23, *p* = 0.4310), with a bias estimate of −2.3228 (SE = 2.8978), suggesting no evidence of significant publication bias (Figure S2, Funnel Plot). The funnel plot asymmetry may be attributable to the substantial between-study heterogeneity (I^2^ = 98.9%) rather than publication bias.

### 2.4. Nucleotide and Deduced Amino Acid Sequence Analysis

#### 2.4.1. Nucleotide and Amino Acid Sequence Analysis of DENV-1

**Figure 2:**
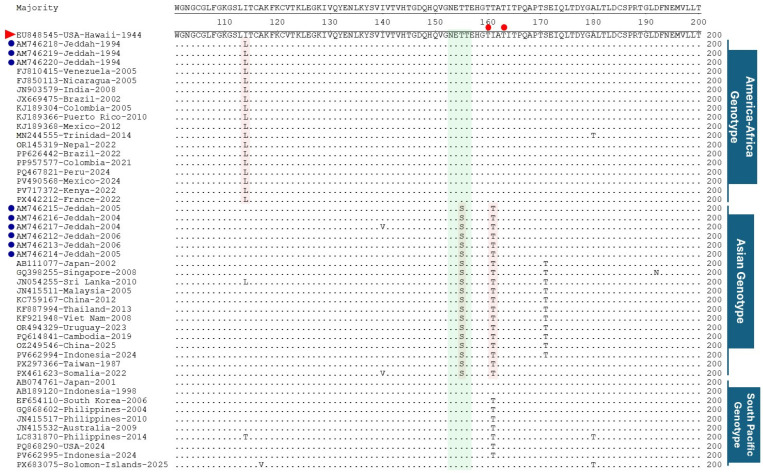
Multiple sequence alignment of the complete *E* protein sequences of DENV-1 from Saudi Arabian isolates (collected between 1994 and 2006) and representative international reference strains. The alignment was performed via the ClustalW method within the MegAlign program (V7.1.0). The prototype strain USA/Hawaii/1944 (GenBank accession EU848545) served as the reference sequence (indicated by a red triangle). Dots (.) represent nucleotides identical to the reference. Major genotypes are indicated on the right: the America–Africa genotype (including Saudi isolates from 1994) and the Asian genotype (including Saudi isolates from 2004 to 2006). Saudi Arabian isolates are highlighted by blue circles on the left. Selected key genotype-specific amino acid positions are highlighted by red rectangles in the figure; the remaining genotype-specific loci are shown in the Supplementary Figures (Figures S3–S6). N-linked glycosylation sites are highlighted by green rectangles, and O-linked glycosylation sites are indicated by red circles.

At the nucleotide sequence level, sequence analysis revealed that the consensus strain, the Hawaii-1944 strain, shares 92.6–94.3% nucleotide sequence identity with Saudi Arabian isolates (1994–2023). The Saudi strains have 98–100% sequence identity, with the 2004–2006 Jeddah isolates showing the highest similarity to the consensus. These findings indicate moderate genetic divergence between the historical Hawaiian lineage and contemporary Saudi Arabian strains. For DENV-1, a total of 110 nucleotide mutations were identified, of which 11 resulted in amino acid changes in strains since 1994. Seven residues (K52N, P227S, E234Q, K277T, N290D, L402F, and A473T) were classified as permanent substitutions, as they were present in all genotypes relative to the consensus sequence. The 1994 isolates contained 73 nucleotide mutations, resulting in nine amino acid changes (D37N, I114L, M297V, A369T, V380I, I439V, A481V). The 2004–2023 isolates contained 106 nucleotide mutations, resulting in 12 amino acid substitutions, of which five (P227S, E234Q, K277T, L402F, A473T) were shared with the 1994 strains, and seven were unique to the later period (T81A, I140V, T155S, I161T, S225T, V300A, A313V, L351V, S397T, I461V, M483L) (Figure 2). Genotype-specific analysis assigned D37N, I114L, A369T, and I439V to the America–Africa genotype; T155S, I161T, I461V, and M484L to the Asian genotype; and N89T and T339S to the South-Pacific genotype. A single substitution, A313V, was unique to Saudi strains isolated between 2004 and 2006 (Figures S3–S6).

#### 2.4.2. Nucleotide and Amino Acid Sequence Analysis of DENV-2

**Figure 3:**
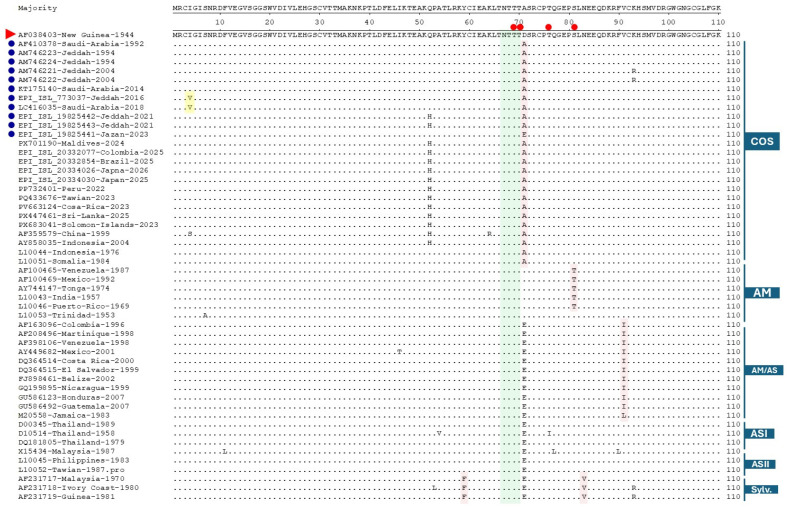
Multiple sequence alignment of the complete *E* protein sequences of DENV-2 from Saudi Arabian isolates (1992–2023) and representative international reference strains. The alignment was performed via the ClustalW method within the MegAlign program (V7.1.0). The prototype strain New Guinea/1944 (GenBank accession AF038403) served as the reference sequence (indicated by a red triangle). Dots (.) represent nucleotides identical to the reference. Major genotypes are indicated on the right: cosmopolitan (COS)—all Saudi isolates; American (AM); American/Asian (AM/AS); Asian (ASI); and sylvatic. Saudi Arabian isolates are highlighted by blue circles on the left. Saudi Arabian isolates are indicated by blue circles on the left. In the figure, key genotype-specific amino acid positions are marked with red rectangles, while amino acids unique to Saudi strains are highlighted with yellow rectangles; the remaining genotype-specific loci are shown in the Supplementary Figures (Figures S7–S10). N-linked glycosylation sites are highlighted by green rectangles, and O-linked glycosylation sites are indicated by red circles.

Sequence analysis revealed that the consensus strain (New Guinea 1944) shares 93.8–95.3% nucleotide identity with Saudi Arabian isolates (1992–2023). The Saudi strains presented strong intragroup similarity (generally 97–100%), particularly among the 1994 Jeddah and 2021 Jeddah isolates. The highest similarity to the consensus strain was observed with the 1992 Saudi Arabian strain (95.3%) and several 1994 Jeddah isolates (≈95.0–95.2%). These results indicate relatively close genetic relatedness between the historical consensus strain and contemporary Saudi Arabian strains. For DENV-2, strains from 1992 to 2018 presented 117 nucleotide mutations and 20 amino acid changes (I4V, I46T, D71A, K93R, K126E, V129I, I141V, H149N, I163V, T171S, E202K, T226I, I322V, T359A/M, E360G, N390S, I402F, V461A, I462V). Strains from 2021 to 2023 presented 157 nucleotide mutations and 22 amino acid substitutions, including novel changes (I6M, K47E, T48K, Q52H, N83S, T120R, I162V, I170V, I443V, N449S, S478T, V484I) and persistent changes (D71A, K126E, V129I, H149N, I163V, N390S, I402F, I462V) (Figure 3). Two substitutions, I402F and K126E, were classified as permanent substitutions, as they were present in all genotypes compared with the consensus sequence. Genotype-specific residues were identified as follows: AM genotype (S81T, I139V, N390D), AM-AS genotype (V91I), COS genotype (D71A, H149N, I164V, N390S, I462V), and sylvatic genotype (Y59F, N83V, K122L, V181I, T236M, R345K, V365I, I432V, S478T). Five unique amino acid changes (I4V, T120R, T359M, E360G, and K361R) were observed specifically in Saudi strains (Figures S7–S10).

#### 2.4.3. Nucleotide and Amino Acid Sequence Analysis of DENV-3

**Figure 4:**
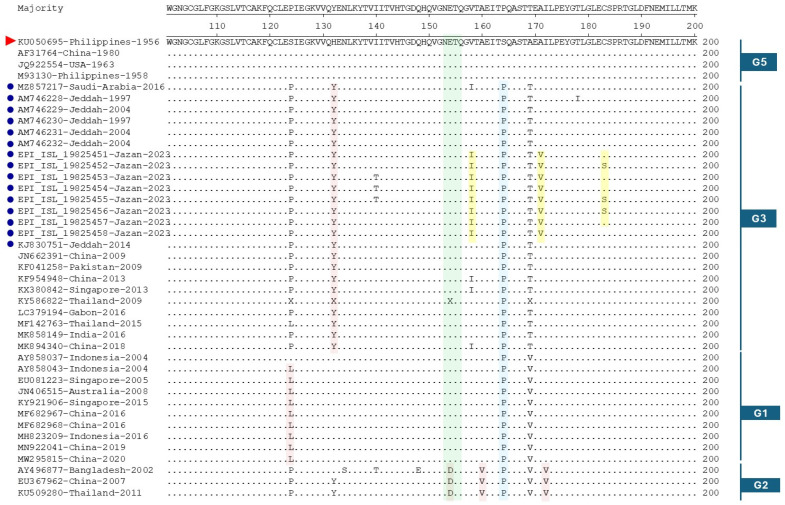
Multiple sequence alignment of the complete *E* protein sequences of DENV-3 from Saudi Arabian isolates (1997–2023) and representative international reference strains. The alignment was performed via the ClustalW method within the MegAlign program (V7.1.0). The prototype strain Philippines/1956 (GenBank accession KU050695) served as the reference sequence (indicated by a red triangle). Dots (.) represent nucleotides identical to the reference. Major genotypes are indicated on the right: Genotype I (G1), Genotype II (G2), Genotype III (G3), and Genotype V (G5). Saudi Arabian isolates (Jeddah 1997–2004 and Jazan 2023) belong exclusively to Genotype III and are highlighted by blue circles on the left. In the figure, key genotype-specific amino acid positions are marked with red rectangles, while amino acids unique to Saudi strains are highlighted with yellow rectangles; the remaining genotype-specific loci are shown in the Supplementary Figures (Figures S11–S14). N-linked glycosylation sites are highlighted by green rectangles. The light blue highlighted rectangle indicates amino acids shared by all genotypes except Genotype 5.

Sequence analysis revealed that the 1956 Philippines strain shared 92.5–93.9% nucleotide identity with Saudi Arabian isolates, corresponding to approximately 6.1–7.5% divergence. Saudi strains clustered tightly together with high intragroup similarity (typically 96.6–99.9%). The highest similarities to the Philippines 1956 reference were observed with the 2016 Saudi Arabian strain (93.9%) and several 1997–2004 Jeddah isolates (≈93.6–93.9%). Overall, the Saudi Arabian strains presented moderate genetic distance from the historical Philippines lineage. The DENV-3 strains from 1997 to 2014 harbored 128 nucleotide mutations resulting in 16 amino acid substitutions (V21I, I81V, S124P, H132Y, S164P, A169T, T178I, T219A, T223I, T270N, K383N, R391K, I452V, T471I, V489A). Strains from 2016 to 2023 presented 119 nucleotide mutations and 17 amino acid changes, sharing ten residues with earlier strains (I81V, S124P, H132Y, S164P, A169T, T270N, K383N, R391K, I452V, V489A) and introducing additional substitutions (I140T, V158I, A171V, E182S, I380T/A, F400L) (Figure 4). Two residues, S164P and R391K, were classified as permanent substitutions, as they were present in all genotypes relative to the consensus sequence. The genotype-specific amino acid residues were as follows: Genotype I (I68V, S124L, R231K, L301S, T303A, and V377I); Genotype II (E154D, A160V, and I172V); Genotype III (I81V, H132Y, and I452V); and Genotype V (E225K). Saudi strains presented five unique amino acid changes: V158I, A171V, C183S, I380A, and F400L (Figures S11–S14).

#### 2.4.4. Predicted O Glycosylation Sites in the *E* Protein of DENV-1, DENV-2, and DENV-3

In DENV-1, Saudi strains of the American–African genotype (Jeddah 1994) consistently presented seven predicted O glycosylation residues (70, 76, 160, 163, 222, 225, 272) (Figures S1–S4). In contrast, all Saudi Asian genotype isolates (Jeddah 2004–2011) displayed an extended pattern that additionally included positions 226, 227, and 230. This genotype specific expansion was stable over a decade. International DENV-1 strains largely reflected this dichotomy: American–African isolates from France, Mexico, Brazil, Kenya, and the Americas retained the seven-site core, whereas Asian genotype strains from Singapore, China, Taiwan, Indonesia, and Vietnam consistently added positions 226 230. Interestingly, some Asian international strains also predicted position 161 (scores ~0.51 0.53), a residue not observed in Saudi Asian isolates. South Pacific genotype strains (Philippines, Solomon Islands, Indonesia) predominantly followed the seven-site pattern, although one Philippine isolate (2014) uniquely displayed low probability sites at 69, 273, and 275. For DENV-2, all Saudi strains belonged to the COS genotype and, from 1992 onward, exhibited a characteristic five-residue pattern (69, 70, 76, 81, 229), with position 76 showing the highest prediction scores (>0.82) (Figures S5–S8). An exceptional Jazan 2023 Saudi isolate lacked residues 69 and 81, retaining only 70, 76, and 229 residues. Internationally, DENV-2 genotypes vary considerably: American (AM) and Asian (ASI) strains frequently predict only the core triad 70 76 229, with 69 and 81 strains appearing sporadically (e.g., Mexico 1992, Colombia 2025, Japan 2025). Sylvatic strains (Guinea 1981, Philippines 1983) also carried the 70 76 pair, whereas the Thai 1958 (ASI) isolate completely lacked position 76, the most conserved DENV-2 site overall. Thus, across the two serotypes, Saudi strains often define genotype-specific reference patterns, against which international strains show either conservation (DENV-1 Asian) or a marked reduction in site number (DENV 2 non COS genotypes).

For DENV-3, all Saudi strains (Genotype 3) from Jeddah (1997–2004) and Jazan (2023) shared a highly stable set of seven predicted residues: 70, 76, 220, 223, 224, 226, and 228 (Figures S9–S12). Position 159 was present only in older Jeddah isolates (1997-2004) but was absent in all Jazan 2023 strains, suggesting a recent temporal loss within the same genotype. International DENV-3 strains revealed clear genotype-dependent profiles. Genotype 1 isolates (Indonesia, Australia, Singapore, China) exhibited the same seven residues but additionally included positions 159 and sometimes 81 (e.g., Singapore 2015, China 2020), indicating a broader pattern than that of Saudi Genotype 3. Genotype 2 strains (China 2007, Thailand 2011, Bangladesh 2002) uniquely featured position 81 with high probability (~0.58) and occasionally lacked residue 76. Genotype 5 isolates (Philippines 1956, USA 1963, China 1980) uniformly retained seven site cores (70, 76, 220, 223, 224, 226, 228) with elevated prediction scores (>0.84) observed specifically at positions 223 and 228 but never included 159 or 81. A Gabon 2016 isolate (unassigned genotype) presented substantially lower prediction probabilities across all sites, indicating possible regional divergence. Overall, the Saudi DENV-3 Genotype 3 pattern (70, 76, 220, 223, 224, 226, 228) was highly conserved across most international genotypes except for the addition of accessory residues (159, 81) in Genotype 1 and the loss of 76 in some Genotype 2 strains. This finding underscores that within each serotype, Saudi strains provide robust reference profiles for genotype-specific O glycosylation, whereas international strains exhibit predictable variation according to their assigned genotype.

#### 2.4.5. Predicted N-Linked Glycosylation Sites in the *E* Protein of DENV-1, DENV-2, and DENV-3

Analysis (0.5698, 7/9, “+”), with no detectable genotype-specific variation. Thus, while the two N-linked sites (67 and 153) are universally conserved across all three serotypes and most genotypes, subtle differences in probability and flanking motifs distinguish DENV-2 genotypes of predicted N-linked glycosylation motifs (Asn-X-Ser/Thr) revealed that all three serotypes consistently possess two conserved sites at positions 67 and 153, albeit with genotype- and strain-specific variations in the surrounding tetrapeptide sequences and prediction probabilities. For DENV-1, both Saudi and international strains (all Asian genotypes) presented an identical pattern: position 67 with the motif NTTT (probability 0.6588, 9/9 strains, “++” confidence) and position 153 with either NEST or NETT (0.5546, 6/9 strains, “+” confidence). No genotype-related differences were observed among the DENV-1 strains. In DENV-2, Saudi strains (all COS genotypes) uniformly displayed position 67 as NTTT (0.6588–0.6744, 9/9, “++”) and position 153 as NDTG (0.5543–0.5546, 6-7/9, “+”), with a minor increase in probability at position 67 for the Jazan-2023 isolate (0.6744). International DENV-2 strains revealed clear genotype-dependent variation: COS genotype isolates from Maldives, Colombia, Brazil, Japan, and Somalia matched the Saudi COS pattern exactly. In contrast, American (AM) genotype strains (Venezuela-1987, Mexico-1992, Tonga-1974, Puerto Rico-1969, Colombia-1996) presented slightly elevated probabilities at positions 67 (0.6801) and 153 (0.5698–0.5700, 7/9, “+”). AM/AS intermediate genotypes (Venezuela-1998, Costa Rica-2000, Nicaragua-1999, Guatemala-2007, Jamaica-1983) presented intermediate probabilities: positions 67~0.6743–0.6746 and positions 153~0.5476–0.5477 (7/9, “+”). The Asian (ASI) and sylvatic strains displayed a mixed pattern, with Thailand-1958 (ASI) having a slightly lower position-67 probability (0.6617, 8/9, “+”). For DENV-3, all Saudi strains (no genotype specified but likely Genotype 3) and all international strains across genotypes uniformly presented position 67 as NVTT or NITT (probability 0.6692, 9/9, “++”) and position 153 as NETQ (COS, AM, AM/AS, ASI), and to a lesser extent, DENV-1 and DENV-3 show remarkable uniformity between Saudi and international strains.

### 2.5. Phylogenetic Analyses of DENV-1, DENV-2 and DENV-3

**Figure 5:**
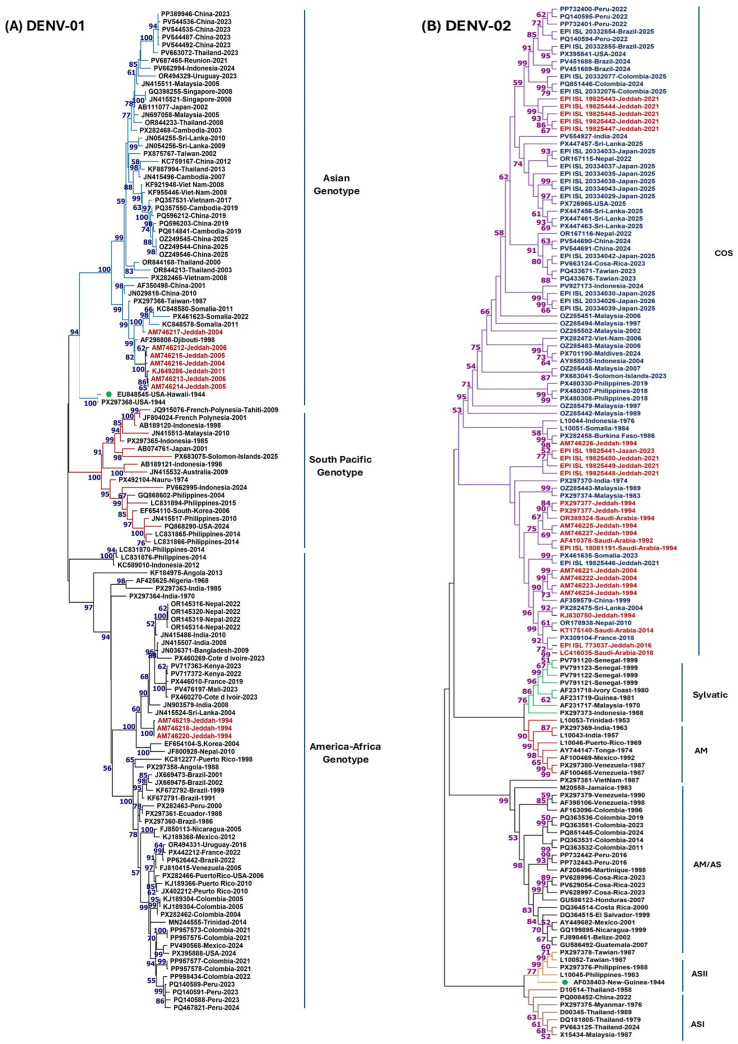

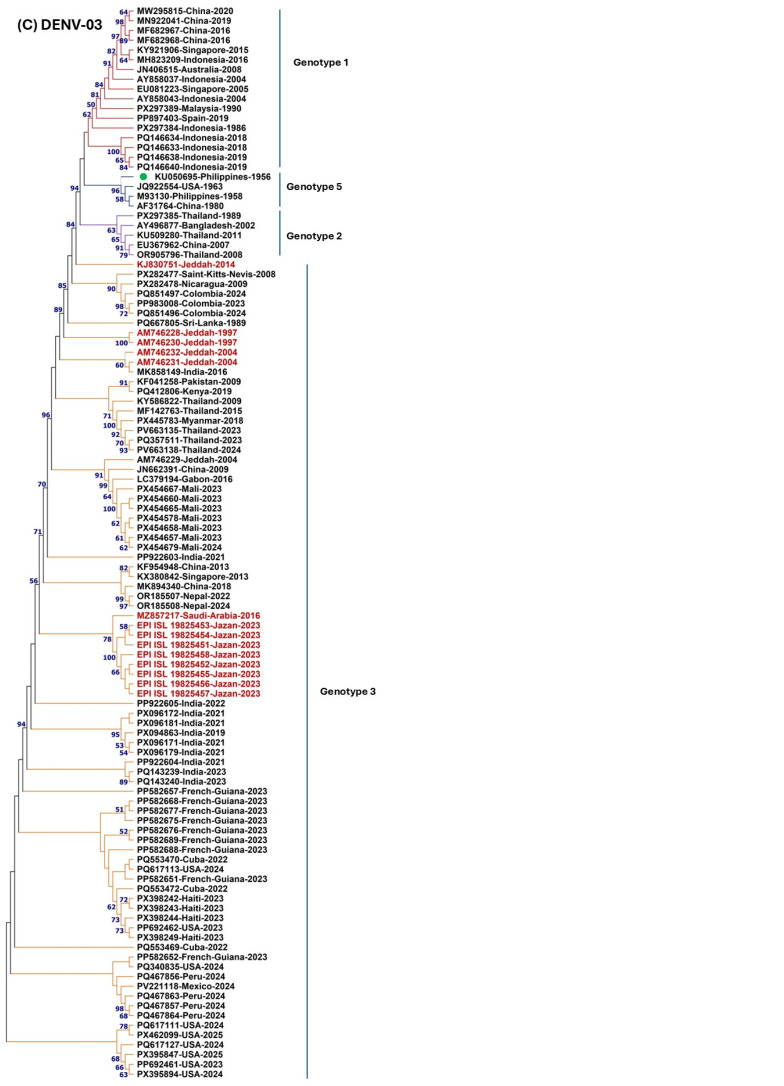
Phylogenetic trees of dengue virus serotypes 1, 2, and 3 based on complete *E* gene sequences. Trees were constructed using maximum likelihood with the best-fit substitution models identified by BIC-based model selection: TN93+G+I for DENV-1, and TN93+G for DENV-2 and DENV-3, with 1000 bootstrap replicates. Bootstrap support values (≥50%) are shown at nodes. Trees are midpoint-rooted. Branch lengths are proportional to genetic distance. (**A**) DENV-1: 129 sequences, 1485 nucleotides. Saudi strains from 1994 (AM746218–AM746220) cluster within the America–Africa genotype (99%), whereas strains from 2004 to 2006 (AM746212–AM746217) and 2011 (KJ649286) belong to the Asian genotype (98%). (**B**) DENV-2: 139 sequences, 1485 nucleotides. All 26 Saudi strains (1992–2023) group within the Cosmopolitan (COS) genotype (100%), clearly separated from the Asian II genotype (99%). (**C**) DENV-3: 110 sequences, 1479 nucleotides. All 14 Saudi strains belong to Genotype III (99%), which includes recent isolates from the Americas (2023–2025). All analyses were conducted in MEGA11 (V10.2.6). Saudi strains are indicated by red font, prototype strains are marked with green circles, and different branch colors represent distinct genotypes as described above.

For DENV-1 (Figure 5A), the tree topology revealed two distinct genotypes among the Saudi strains. Three Jeddah strains from 1994 (AM746218, AM746219, AM746220) grouped within the American–African genotype with strong bootstrap support (99%), clustering with strains from Somalia, Djibouti, and other African/European lineages. In contrast, six Jeddah strains collected between 2004 and 2006 (AM746212–AM746217) and one from 2011 (KJ649286) clustered with the Asian genotype (bootstrap = 98%), showing close genetic relationships to strains from Singapore, China, Thailand, Taiwan, and Vietnam. The separation between the American–African and Asian genotypes was strongly supported (bootstrap = 100%), indicating a clear genotype replacement event in the early 2000s. The remaining sequences (mainly from Peru, 2023) formed a single, highly homogeneous clade, consistent with a recent outbreak. The overall DENV-1 phylogeny indicates that Saudi Arabia has experienced multiple introductions from both American–African and Asian genotypes over time, with the Peruvian 2023 outbreak representing a genetically uniform lineage.

For DENV-2 (Figure 5B), all 26 Saudi strains spanning the years 1992–2023 were grouped exclusively within the COS genotype, forming a monophyletic clade with strong bootstrap support (100%). The tree showed a well-resolved structure with the COS genotype clearly separated from the Asian II genotype (bootstrap = 99%), confirming the genetic distinctiveness of these lineages. The Saudi isolates showed excellent internal structure: the 1992 isolate (AF410378) occupied the deepest branching position, followed by 1994 isolates (AM746221–AM746227), while isolates from 2004 to 2023 formed a more recent, tightly clustered subclade. Notably, the 2023 Jazan isolates showed close genetic relationships to 2021 Jeddah isolates (short branch lengths), suggesting recent regional transmission. The scale bar (0.02 substitutions per site) indicates a small but consistent genetic distance within the COS clade. This result confirms that the COS genotype has been the predominant DENV-2 lineage circulating in Saudi Arabia for nearly three decades, whereas other genotypes (e.g., Asian I, American) were present in the global dataset but not detected among the Saudi isolates.

For DENV-3 (Figure 5C)**,** all 14 Saudi sequences (Jeddah 1997–2004, Jazan 2023, and one from 2016) belonged exclusively to Genotype III, forming a well-supported clade (bootstrap = 99%). The global DENV-3 phylogeny showed deep branching, with historical strains from the Philippines (1956, 1958) and the USA (1963) occupying basal positions within Genotype V. A strongly supported major clade (bootstrap = 99%) contained recent isolates from the Americas (Peru, Mexico, USA; French Guiana, Cuba; 2023–2025) together with Saudi strains and Indian isolates (2021–2023). The Saudi isolates formed a tight cluster with very short branch lengths, indicating recent local circulation and close genetic relationships. This pattern indicates that Genotype III has been the dominant lineage globally and that the Saudi isolates are part of a broader intercontinental clade that also seeded recent outbreaks in the Western Hemisphere.

### 2.6. Predominance of Purifying Selection with Episodic Adaptive Signals in DENV Serotypes S

Selection pressure analysis was performed on the DENV *E* gene for all three serotypes (DENV-1, DENV-2, and DENV-3) (Table 2) using four complementary approaches (SLAC, FEL, FUBAR, and MEME) to identify sites under pervasive and episodic selection (Table 2). SLAC identified 172 negatively selected sites and no positively selected sites. FEL confirmed these findings, detecting approximately 275 negatively selected sites and no positively selected sites. FUBAR analysis revealed that all 495 codons showed strong evidence of negative selection (posterior probability ≥ 0.9), with no sites showing evidence of positive selection. MEME detected episodic positive selection at two sites: Site 82 (*p* = 0.00) and Site 327 (*p* = 0.00), with extremely high ω values on specific branches (β^+^ = 6609.38 and 1070.23, respectively), indicating strong adaptive evolution on particular lineages.

In the case of DENV-2, FEL detected 22 positively selected sites (*p* < 0.1), indicating evidence of pervasive positive selection. SLAC identified approximately 150 negatively selected sites, while FUBAR confirmed that all 495 codons showed strong evidence of negative selection. MEME detected episodic positive selection at three sites: Site 202 (*p* = 0.04), Site 404 (*p* = 0.00), and Site 493 (*p* = 0.00). Sites 404 and 493 exhibited extremely high β^+^ values (100,000 and 10,627.80, respectively), suggesting very strong episodic selection on specific branches.

For the DENV-3, SLAC and FEL identified approximately 120 and 100 negatively selected sites, respectively, with no sites showing pervasive positive selection. FUBAR confirmed that all 568 codons were under strong purifying selection. MEME detected episodic positive selection at five sites: 96 (*p* = 0.07), 207 (*p* = 0.07), 209 (*p* = 0.11), 215 (*p* = 0.08), and 455 (*p* = 0.01). Site 455 showed the strongest signal (β^+^ = 9.76), suggesting immune-driven adaptation on specific viral lineages.

## 3. Discussion

The first documented dengue outbreak in Saudi Arabia occurred in Jeddah in 1994, with 289 confirmed cases involving co-circulation of DENV-1 and DENV-2, an event that marked the establishment of the virus in the western region [9,17,38]. Since then, recurrent outbreaks have spread to Makkah, Al-Madinah, Jizan, and Najran, leading to the declaration of the western region as dengue-endemic in 2004 [12,13,14,27]. Our epidemiological synthesis confirms that DENV-2 has remained the predominant serotype across most regions and time periods, followed by DENV-1 and DENV-3, while DENV-4 remains rare—a pattern consistent with previous systematic reviews [12,15] and recent hospital-based studies [18,33]. The demographic profile—male predominance (67–78%) and young adult susceptibility (25–44 years)—is consistent with global observations and likely reflects occupational exposure (outdoor work, farming) and cultural practices [13,18,31,42,43]. The seasonal bimodal pattern (spring and winter peaks) correlates with temperature, humidity, and rainfall patterns that favor *Aedes* mosquito breeding, as well as the timing of pilgrimage activities [44,45,46,47].

The co-circulation of DENV-1, DENV-2, and DENV-3 in Saudi Arabia is of major public health concern, as it fundamentally increases the population’s risk for severe dengue (DHF/DSS) through secondary heterotypic infections [9,17]. A case–control study from Jeddah confirmed that secondary infection is a significant risk factor for severe disease in the Kingdom (*p* = 0.02) [31], while viral serotype was not found to be a significant predictor of severity in that cohort. Globally, the risk of severe disease is known to vary by serotype and infection history, with secondary DENV-2 and primary DENV-3 infections often associated with higher severity. In the Saudi context, the predominance of DENV-2 (80.25% in the 2023–2024 Makkah cohort) [18] is particularly concerning given the association of this serotype with more severe outcomes in secondary infections [48]. However, the Jeddah study found that host factors such as comorbidities (diabetes, hypertension) and secondary infection status were stronger predictors of severe dengue than serotype alone [31]. These findings highlight the need for enhanced surveillance of severe cases stratified by serotype and infection history to better define risk groups and inform clinical management in the Kingdom.

Our phylogenetic analyses of DENV *E* gene sequences from Saudi Arabia (1994–2023) provide critical insights into the evolutionary dynamics and introduction histories of circulating serotypes. It is important to note that these phylogenetic and molecular findings primarily reflect the situation in the western provinces (Jeddah, Makkah, Jazan), where the vast majority of cases and all available sequenced isolates originate. For DENV-1, we observed a clear temporal shift in genotype composition: all three 1994 Jeddah isolates (AM746218, AM746219, AM746220) clustered within the American–African genotype (bootstrap = 99%), whereas six isolates from 2004 to 2006 (AM746212–AM746217) and one from 2011 (KJ649286) grouped within the Asian genotype (bootstrap = 98%). The separation between these two genotypes was strongly supported (bootstrap = 100%). This finding suggests a genotype replacement event in the early 2000s, likely driven by repeated introductions via expatriate workers and pilgrims from dengue-endemic Asian countries [9,13,17]. Such genotype shifts have been associated with changes in disease severity and transmission dynamics in other endemic regions [49,50,51]. The robust bootstrap support (>98%) for both genotype assignments in our model-based ML analysis (TN93+G+I) confirms that these patterns are not artifacts of the analytical method.

In contrast, all 26 Saudi DENV-2 isolates spanning nearly three decades (1992–2023) belonged exclusively to the COS, forming a monophyletic clade with strong bootstrap support (100%). The COS genotype was clearly separated from the Asian II genotype (bootstrap = 99%), confirming the genetic distinctiveness of these lineages. This remarkable stability—with the 1992 isolate occupying the deepest branching position, followed by 1994 isolates, and isolates from 2004 to 2023 forming a more recent subclade—could reflect local adaptation to the local vector and environmental conditions. However, alternative explanations are equally plausible: a founder effect from a single introduction that subsequently expanded locally, or repeated introductions of closely related COS strains from the global gene pool. Our data cannot distinguish among these scenarios; distinguishing them would require whole-genome phylogeographic analyses and nucleotide diversity metrics. Therefore, the interpretation of COS dominance as evidence of local adaptation should be considered one of several plausible explanations rather than a definitive conclusion. Notably, the 2023 Jazan isolates showed close genetic relationships to 2021 Jeddah isolates (short branch lengths), suggesting recent regional transmission. The COS genotype of DENV-2 has been associated with widespread global dispersal and has replaced other genotypes in several regions [52,53,54,55]. Our results, derived from rigorous model-based ML analysis (TN93+G), corroborate previous reports of DENV-2 COS dominance in Jeddah, Makkah, and Jizan [17,20,56] and extend the temporal range of this dominance to include the 2023 Jazan isolates.

For DENV-3, all 14 Saudi isolates (Jeddah 1997–2004, Jazan 2023, and one from 2016) belonged exclusively to Genotype III, forming a well-supported clade (bootstrap = 99%). This clade included recent isolates from the Americas (Peru, Mexico, USA, French Guiana, Cuba; 2023–2025) and Indian isolates (2021–2023). The topology indicates that Saudi Genotype III strains are part of a broader intercontinental lineage that has also seeded recent outbreaks in the Western Hemisphere. The deep basal position of historical strains from the Philippines (1956, 1958) and the USA (1963) within Genotype V is consistent with the known global spread of DENV-3 Genotype III from Southeast Asia to the Americas and the Middle East [57,58]. The Saudi isolates formed a tight cluster with very short branch lengths, indicating recent local circulation and close genetic relationships. Our model-based ML analysis (TN93+G) confirms that Genotype III has been the exclusive lineage of DENV-3 circulating in Saudi Arabia.

Collectively, these results demonstrate three contrasting evolutionary and epidemiological patterns across serotypes: DENV-1 shows historical co-circulation of American–African and Asian genotypes with a genotype replacement event in the early 2000s; DENV-2 is dominated by a single cosmopolitan lineage spanning three decades; and DENV-3 is exclusively represented by Genotype III, which has recently expanded into the Americas. The use of rigorous model-based approaches (TN93+G+I for DENV-1; TN93+G for DENV-2 and DENV-3) with strong bootstrap support at key nodes provides robust phylogenetic inference across all three serotypes.

We identified several permanent amino acid substitutions across all three serotypes that distinguish circulating Saudi strains from prototype reference strains. In DENV-1, seven permanent changes (K52N, P227S, E234Q, K277T, N290D, L402F, A473T) were present in both the 1994 and 2004–2023 isolates. Similarly, DENV-2 presented two permanent substitutions (K126E, I402F), and DENV-3 presented two (S164P, R391K). Some of these residues are located in functionally important domains of the *E* protein. For example, position 402 (in both DENV-1 and DENV-2) lies in the stem–anchor region involved in membrane fusion and virion assembly [59,60], while position 277 in DENV-1 falls within domain III, a major target of neutralizing antibodies [61,62,63]. Several Saudi-unique amino acid changes were also identified (e.g., A313V in DENV-1 and others in DENV-2 and DENV-3). The functional significance of these substitutions requires further experimental validation through in vitro and in vivo studies.

Selection pressure analysis revealed that the DENV *E* gene is predominantly under strong purifying selection across all three serotypes, consistent with its critical role in viral entry and membrane fusion [64,65]. However, notable serotype-specific differences were observed: DENV-2 exhibited pervasive positive selection (22 FEL sites, *p* < 0.1), unlike DENV-1 and DENV-3, suggesting stronger immune-driven adaptation in this serotype [65,66]. Episodic positive selection was detected in all three serotypes using MEME, with DENV-3 showing the highest number of episodic sites (*n* = 5), indicating that immune-driven adaptation occurs at specific sites on particular lineages rather than across the entire phylogeny [65]. Among the permanent amino acid substitutions identified, only two were under purifying selection: I402F in DENV-2 (located in domain III, critical for receptor binding) and S164P in DENV-3 (located in domain II, containing the fusion loop) [67,68]. The seven permanent substitutions in DENV-1, as well as K126E in DENV-2 and R391K in DENV-3, were not under purifying selection, suggesting neutral fixation through genetic drift rather than functional constraint [69]. The detection of pervasive positive selection in DENV-2, coupled with its epidemiological predominance and association with severe disease [48], suggests that this serotype may have a higher capacity for immune escape, with important implications for vaccine design and genomic surveillance.

Our glycosylation prediction analysis revealed interesting temporal and genotype-specific patterns. All DENV-1 Asian genotype strains (2004–2011) acquired three additional predicted O glycosylation sites (226, 227, 230) compared with the earlier American–African genotype (1994), which possessed only the core seven sites (70, 76, 160, 163, 222, 225, 272). These emergent sites cluster in domain II of the E protein, which is involved in membrane fusion and antibody recognition [70,71]. The acquisition of additional O glycans may alter protein folding, stability, or immune recognition [72] and could represent an adaptive shift accompanying genotype replacement. In contrast, DENV-2 COS genotype isolates presented a stable five-residue pattern (69, 70, 76, 81, 229), with an exceptional Jazan 2023 isolate lacking residues 69 and 81. The DENV-3 Genotype III isolates displayed a conserved seven-site pattern (70, 76, 220, 223, 224, 226, 228), although position 159 was present in older Jeddah isolates (1997–2004) but absent in all Jazan 2023 strains, suggesting recent temporal loss. N-linked glycosylation sites at positions 67 and 153 are universally conserved across all serotypes and genotypes, which is consistent with their critical role in protein folding, virion stability, and receptor binding [72,73]. It is important to emphasize that the O-glycosylation sites identified in this study are based entirely on computational predictions. To date, no experimental validation (e.g., such as mass spectrometry, lectin binding assays, or site-directed mutagenesis) has been performed to confirm the presence of O-linked glycans at these specific residues on dengue virus *E* proteins derived from Saudi isolates. Therefore, the functional implications of these predicted sites (e.g., effects on folding, stability, or immune recognition) remain hypothetical and require experimental confirmation.

Despite serological and PCR evidence of DENV-4 circulation in Saudi Arabia [16,18], no complete or partial *E* gene sequences from DENV-4 derived from Saudi human cases are available in GenBank or GISAID. This absence is not unexpected given the extremely low prevalence of DENV-4, which naturally results in very few positive samples available for sequencing. This relative absence may stem from several factors: (i) the very low circulation of DENV-4, leading to fewer positive samples available for sequencing; (ii) prioritization of sequencing efforts toward the dominant serotypes (DENV-1–3) in resource-limited surveillance settings; (iii) logistical and technical barriers, including limited next-generation sequencing capacity and delays in data submission to public repositories; and (iv) possible focus on local diagnostic rather than research-oriented sequencing. It is also plausible that unpublished datasets exist in national or institutional repositories.

In regional countries with strong migratory and pilgrimage links to Saudi Arabia, such as Pakistan and India, DENV-4 sequences are more readily available. Pakistan routinely reports co-circulation of all four serotypes with deposited DENV-4 genomes [24,74], while India has documented DENV-4 circulation with multiple genotypes, including Genotype I [75]. Yemen has also reported all four serotypes, with genomic data available for related strains [76,77]. While DENV-4 remains a minor serotype in Saudi Arabia, the complete lack of genomic data—even for the few detected cases—still represents a missed opportunity for molecular surveillance. This contrast highlights the value of sequencing low-prevalence serotypes to better understand cross-border transmission dynamics via pilgrims and expatriates.

To address this gap, we recommend a targeted surveillance framework: (1) routine whole-genome or E-gene sequencing of all DENV-positive samples regardless of serotype, with a minimum quota for low-prevalence serotypes such as DENV-4; (2) integration of genomic surveillance into the national Ministry of Health dengue program, including incentives for timely deposition into GISAID/GenBank; (3) capacity building through partnerships with regional centers (e.g., in Pakistan or UAE) for sequencing support; and (4) periodic retrospective sequencing of archived DENV-4 positive samples from blood banks and hospitals. Such measures would enable genotype identification, monitoring of importation events, and assessment of whether DENV-4 is maintained through cryptic local transmission or repeated introductions.

Our findings have several practical implications. The predominance of the DENV-2 cosmopolitan genotype and the temporal shift in DENV-1 from America–Africa to Asia highlight the need for continuous molecular surveillance to detect emerging lineages that may be associated with increased virulence or epidemic potential [49,56]. The identification of Saudi unique amino acid substitutions and genotype-specific glycosylation patterns warrants further investigation via reverse genetics and in vitro assays to assess their impact on viral fitness, antibody neutralization, and vaccine efficacy. The recent availability of dengue vaccines such as TAK-003 (Qdenga) and Dengvaxia underscores the importance of continued molecular surveillance of circulating genotypes and serotypes in Saudi Arabia, as vaccine performance can vary by serotype and genotype. Currently approved dengue vaccines have demonstrated variable efficacy depending on serotype and prior exposure status. Notably, the predominant DENV-2 Cosmopolitan genotype identified in Saudi Arabia shares the same serotype as the DENV-2 component in TAK-003; however, the degree of antigenic match between the Saudi Cosmopolitan strains and the vaccine strains remains unknown due to the absence of antigenic cartography or cross-neutralization studies using local isolates. Future studies incorporating serological neutralization assays and antigenic characterization of Saudi DENV-2 strains against vaccine reference strains will be essential to evaluate potential vaccine effectiveness in the local epidemiological context. Until such data are available, reliance on molecular surveillance data alone is insufficient for definitive vaccine recommendations [61,69,70]. Integrated vector control increased diagnostic capacity, and genomic surveillance should be prioritized in high-burden regions (Jeddah, Makkah, Jazan) and extended to emerging hotspots (Riyadh). The role of mass gatherings (Hajj, Umrah) and expatriate worker mobility in introducing new strains should be addressed through pre-travel screening, post-arrival surveillance, and real-time genomic data-sharing platforms such as GISAID. Recent predictive modeling in Jeddah has shown that temperature, humidity, and pilgrimage variables can forecast dengue incidence with good accuracy [44], supporting proactive, data driven outbreak preparedness.

This study has several limitations. First, the available sequence data are heavily skewed toward the western region of Saudi Arabia (primarily Jeddah, Makkah, and Jazan), with little or no *E* gene sequences available from other regions, including Riyadh, the Eastern Province, or northern areas. Consequently, the phylogenetic and molecular findings presented in this study largely reflect the evolutionary dynamics of DENV in the long-established endemic western provinces rather than the country as a whole. Moreover, the 50 isolates analyzed represent all publicly available DENV *E* gene sequences from Saudi Arabia, which constitute only a tiny fraction of the total case burden (thousands of cases annually). These sequences are not derived from a systematic, population-based sampling strategy; rather, they reflect opportunistic sampling during outbreak periods and targeted surveillance in high-burden areas, introducing a risk of selection bias. Extrapolation of these results to non-western regions should be made with caution. Second, the temporal coverage is uneven, with fewer sequences available after 2011 for DENV-1 and after 2021 for DENV-2. Third, the absence of DENV-4 sequences—despite serological evidence of its circulation—precludes any phylogenetic inference for this serotype and further highlights the gaps in current surveillance. Fourth, our analyses relied exclusively on the *E* gene sequences (~1485 nt, approximately 13.5% of the ~11 kb DENV genome). While the *E* gene is a robust phylogenetic marker for serotype and genotype classification, inferences regarding multiple introductions, DENV-1 genotype substitutions, and fine-scale resolution within the DENV-2 Cosmopolitan lineage should be interpreted with caution, as single-gene analyses may miss recombination events or subtle evolutionary signals only detectable by whole-genome sequencing. Finally, potential sampling and submission biases in public databases may not fully represent the true genetic diversity of circulating DENV strains in Saudi Arabia. These limitations underscore the urgent need for expanded, systematic, and geographically representative genomic surveillance across all regions of the Kingdom to obtain a more complete picture of DENV diversity and evolutionary dynamics.

## 4. Materials and Methods

### 4.1. Systematic Review and Meta-Analysis Methodology

This systematic review and meta-analysis was conducted following the PRISMA 2020 guidelines [78]. A systematic literature search was performed across four electronic databases (PubMed, Scopus, Web of Science, and Google Scholar) from January 2003 to April 2026. The search strategy focused solely on DENV in Saudi Arabia. No language restrictions were applied. DENV Studies in Saudi Arabia were included if they met the following criteria: (1) observational studies (cross-sectional, cohort, or case–control) with a minimum sample size of 25 participants; (2) conducted in Saudi Arabia and published between January 2003 and April 2026; and (3) reported primary data on DENV prevalence based on detection of at least one of four biological markers (DENV-NS1, DENV-RNA, anti-DENV IgM, or IgG). Studies conducted outside Saudi Arabia, clinical trials, reviews, case reports, conference abstracts, and studies reporting other diseases were excluded. Titles and abstracts were screened to identify potentially eligible studies. Full texts of selected articles were assessed against the inclusion criteria.

### 4.2. Quality Assessment and Statistical Analysis

Study quality was assessed using the Joanna Briggs Institute (JBI) Critical Appraisal Tool for prevalence studies. The tool consists of nine items assessing internal and external validity, with each item scored as “yes,” “no,” “unclear,” or “not applicable.” Studies were categorized as high-quality (low risk of bias: ≥70% “yes”), moderate-quality (moderate risk: 50–69% “yes”), or low-quality (high risk: ≤49% “yes”).

Meta-analysis was performed using the meta and metafor packages in R (version 4.6.0) [17]. The pooled seroprevalence of DENV infection was calculated using a random-effects model with the DerSimonian–Laird method, reported as proportions with 95% confidence intervals (CIs). Heterogeneity was assessed using the I^2^ statistic, τ^2^, and Cochran’s Q test, with I^2^ values of 25%, 50%, and 75% indicating low, moderate, and high heterogeneity, respectively. Subgroup analyses were performed by geographic region (Jazan, Makkah, Jeddah, etc.), diagnostic assay (IgG, IgM, NS1, RNA/PCR), time period (2003–2010, 2011–2018, 2019–2026), and population type (febrile, healthy, blood donors, etc.). Publication bias was assessed visually using funnel plots and statistically using Egger’s regression test. A *p*-value < 0.05 was considered statistically significant.

### 4.3. Sequence Data Retrieval and Processing

All available DENV *E* gene sequences derived from human cases in Saudi Arabia (1992–2023) were retrieved from the National Center for Biotechnology Information (NCBI) GenBank and the Global Initiative on Sharing All Influenza Data (GISAID) platform. Reference prototype strains were also downloaded for comparative purposes: for DENV-1 (Hawaii 1944 strain: EU848545), DENV-2 (New Guinea 1944 strain: AF038403), and DENV-3 (Philippines 1956 strains: KU050695). Sequence datasets were curated by excluding duplicate entries, short fragments (<500 nt), and sequences with high proportions of ambiguous bases. The raw sequences were edited and assembled via BioEdit software (version 7.0). Multiple sequence alignments for each serotype were initially performed via the Clustal W algorithm implemented in the MegAlign program (DNASTAR Lasergene V7.1). The alignments were manually inspected and adjusted as needed.

A comprehensive search for DENV-4 *E* gene sequences originating from Saudi Arabia was conducted in GenBank and GISAID up to April 2026 using multiple keyword combinations. The search in GenBank returned 3773 results. When filtering specifically for “Saudi Arabia” and “DENV-4” (or “DENV4”), GISAID returned zero sequences. After rigorous screening of all retrieved entries, zero sequences met the inclusion criteria. Sequences were excluded primarily because they were of non-Saudi origin.

### 4.4. Nucleotide and Amino Acid Sequence Analysis

Nucleotide sequences of the DENV *E* gene and their deduced amino acid sequences from Saudi Arabian isolates were analyzed to determine pairwise percent identities and genetic divergence. Saudi sequences were compared against serotype-specific international reference strains retrieved from GenBank, using the respective prototype strains as consensus sequences. Mutations and amino acid substitutions were identified and categorized as permanent (shared across all time periods), time-specific (restricted to 1994 or 2004–2023 isolates), genotype-specific, or potentially Saudi-unique. All sequences used in this study are publicly available in GenBank/GISAID; accession numbers are listed in the Supplementary Materials (Tables S7–S9). To predict potential glycosylation sites in the *E* protein, N-glycosylation motifs were identified via the NetNGlyc 1.0 server (https://services.healthtech.dtu.dk/services/NetNGlyc-1.0/) (accessed on 5 March 2026), and O-glycosylation sites were predicted via the NetOGlyc 4.0 server (http://www.cbs.dtu.dk/services/NetOGlyc/) (accessed on 5 March 2026), both with default thresholds (0.5 for N-glycan and 0.5 for O-glycan predictions). Only residues with prediction scores above the respective thresholds were considered positive. O-glycosylation predictions were cross-validated using the GlycoEP server with only sites predicted by both tools considered high-confidence [79]. Conserved N-glycosylation sites were defined as those present in all analyzed sequences, while O-glycosylation patterns were compared across isolates from different years to identify temporal changes.

### 4.5. Phylogenetic Analysis and Genotype Assignment

To determine the optimal nucleotide substitution model for phylogenetic reconstruction, we performed model selection using the Bayesian Information Criterion (BIC) in MEGA11 (V10.2.6) [80] for each serotype independently. For the DENV-1 dataset, the best-fit model was TN93+G+I (BIC = 25,784.315), with a gamma distribution shape parameter of 1.6037 and 31.14% invariant sites. For DENV-2, the best-fit model was TN93+G (BIC = 33,800.903), with a gamma shape parameter of 0.2742 and no invariant sites. For DENV-3, the best-fit model was TN93+G (BIC = 24,261.233), with no invariant sites. Phylogenetic trees were constructed via the maximum likelihood (ML) method in MEGA11 [80] using these best-fit models with 1000 bootstrap replicates [3] to assess nodal support. Initial trees for the heuristic search were obtained automatically by applying the Maximum Parsimony method, followed by Nearest-Neighbor-Interchange (NNI) branch swapping. All positions containing gaps, missing data, or ambiguous bases were excluded using the partial deletion option with a 95% site coverage cutoff, meaning that positions with less than 95% coverage across all sequences were eliminated. Codon positions were treated as follows: 1st, 2nd, and 3rd positions were included, while noncoding sites were excluded from the analysis. The final datasets comprised 1485 nucleotides for DENV-1 (129 sequences), 1485 nucleotides for DENV-2 (139 sequences), and 1479 nucleotides for DENV-3 (110 sequences). All trees were rooted using the midpoint rooting method in MEGA11, which places the root at the midpoint of the longest branch between two taxa—a widely accepted approach for viral phylogenies when a suitable outgroup is not available. To ensure phylogenetic inference was not confounded by recombination events, we screened the alignments using RDP4; no significant recombination signals were detected among the Saudi isolates, confirming that standard phylogenetic methods are appropriate. Bootstrap support values were interpreted as follows: ≥70% = well-supported; 50–69% = moderately supported; <50% = weakly supported (branches with <50% support were collapsed in the consensus trees). All analyses were conducted in MEGA11.

### 4.6. Selection Pressure Analysis

Selection pressure analysis was performed using four complementary approaches implemented in the HyPhy package (V2.5.100) [81] via the Datamonkey web server [82]. Single Likelihood Ancestor Counting (SLAC) and Fixed Effects Likelihood (FEL) were used to detect pervasive positive and negative selection at individual codon sites, with a significance threshold of *p* < 0.1 [83]. Fast Unconstrained Bayesian AppRoximal (FUBAR) was used to estimate site-specific selection using a Bayesian approach, with a posterior probability ≥ 0.9 considered significant [84]. The Mixed Effects Model of Evolution (MEME) was employed to detect episodic positive selection acting on a subset of branches, with *p* < 0.1 considered significant [84]. All analyses were conducted on the DENV *E* gene dataset for each serotype (DENV-1, DENV-2, and DENV-3) independently, with codon site numbering based on the respective reference genomes. The *E* gene alignment consisted of 495, 560, and 568 codons for DENV-1, DENV-2, and DENV-3, respectively.

### 4.7. Ethical Statement

This study was based exclusively on publicly available genomic sequences and previously published, anonymized epidemiological data. Therefore, no ethical approval or informed consent was needed.

## 5. Conclusions

This comprehensive study integrates three decades of epidemiological, serological, and molecular data to provide the most detailed characterization to date of the evolutionary and transmission dynamics of DENV in Saudi Arabia. Our findings confirm that dengue has become firmly established as a major public health challenge in the western endemic regions of the Kingdom, with a pooled seroprevalence of 30.8%, sustained predominance of DENV-2 (particularly the Cosmopolitan genotype), and co-circulation of DENV-1 (America–Africa and Asian genotypes) and DENV-3 (Genotype III) in these areas. The identification of multiple permanent amino acid substitutions in the *E* protein, genotype-specific and Saudi-unique mutations, and dynamic changes in predicted O-glycosylation patterns highlight ongoing viral adaptation that may influence fitness, antigenicity, and immune evasion in the local ecological niche. These results demonstrate that the western region of Saudi Arabia functions as both a recipient and potential regional hub for DENV introductions, driven by massive expatriate inflows, religious pilgrimages (Hajj and Umrah), and climatic suitability for *Aedes* vectors. The observed genotype replacements (particularly in DENV-1) and the long-term stability of the DENV-2 Cosmopolitan lineage emphasize the need for continuous, high-resolution genomic surveillance to detect emerging variants with altered transmissibility or severity. Strengthening molecular surveillance, expanding whole-genome sequencing capacity across all regions (including emerging central areas such as Riyadh), and ensuring timely deposition of sequences into public databases are essential priorities. Such efforts will be critical for tracking importation events, anticipating outbreaks, evaluating vaccine suitability against locally circulating serotypes and genotypes, and informing targeted vector control and public health interventions. Ultimately, proactive genomic and epidemiological intelligence will be key to mitigating the growing dengue burden in Saudi Arabia and safeguarding population health amid increasing globalization and climate pressures.

## Figures and Tables

**Table 1:** Summary of demographic, serological, and serotype characteristics of the 25 studies included in the systematic review and meta-analysis.

Region	Year	Study Design	Sample Size/Cases	Key Demographic Findings	Laboratory Analysis	Dominant Serotypes	Main Notes	Ref.
Jeddah	2006	Prospective Study	80 suspected, 39 confirmed	Male (3.3:1), Mean age 27.6 years; Mostly non-Saudi	48.75% confirmed	Not specified	Most cases in summer; Main symptoms: fever, myalgia; Lab findings: thrombocytopenia (79.5%), leukopenia (48.7%)	[28]
Jeddah	2008	Cross-sectional	305	Hospitalized patients	53.1% total seroprevalence; 18.4% IgM + IgG; 29.2% PCR+	DENV-3 (59.6%), DENV-2 (22.5%), DENV-1 (17.9%)	High seroprevalence and low incidence detected; RT-PCR recommended over conventional methods	[29]
Makkah	2009	Retrospective Study	159	Male predominance; Mean age 25.6 years; Mostly Saudi (67%)	100% of admitted patients positive by IgM/RT-PCR	Not specified	Most cases in spring/early summer (77.4%); Clinical features: fever, headache, body aches; Low mortality rate (0.6%)	[30]
Jeddah	2012	Case–control	129 cases, 240 controls	Older age (OR = 1.2) was a risk factor	Cases were confirmed by lab investigations	Not specified	Risk factors: stagnant water (OR = 4.9), indoor larvae (OR = 2.2), construction sites (OR = 2.2); Health education decreased risk	[31]
Aseer & Jizan	2013	Cross-sectional	965	Age ≥ 20 years; Male gender	31.7% IgG positive	Not specified	Risk factors: lack of electricity, having water basins in the house	[25]
Jizan	2014	Cross-sectional	553 suspected, 264 confirmed	Saudi (81.44%); Male (70.45%); Age 3–56 years	47.74% positivity; NS1 (25.38%), IgM (26.98%), both NS1 + IgM (33.95%)	Not specified	Highest cases in April (27.69%), lowest in August (0.75%)	[32]
Aseer & Jizan	2014	Cross-sectional	965	Mostly male (69.6%); Adults (20–59 years)	0.8% IgM positive	Not specified	First report of indigenous DENV infection in Aseer; All positive cases were subclinical	[33]
Al-Madinah	2014	Cross-sectional	351 febrile, 1227 afebrile	Middle-aged adults most affected	16.5% IgG in all samples; 18.8% IgM in febrile group	DENV-2 (11 cases), DENV-1 (7 cases)	First study in Al-Madinah; All acute cases were imported	[34]
Makkah	2015	Cross-sectional	100 blood donors	All male (due to cultural barriers); Healthy/eligible donors	1% NS1, 6% IgM, 7% IgG positive	Not specified	High seroprevalence suggests potential risk of transmission through blood transfusion	[35]
Jeddah	2016	Cross-sectional (Community-based)	1939	Male (53%), increasing with age; Mostly asymptomatic	47.8% IgG positive	Not specified	0.1% reported a history of dengue, indicating high asymptomatic rate	[36]
Makkah	2017	Cross-sectional	910 blood donors	All male (due to cultural barriers)	39% IgG, 5.5% IgM positive	DENV-2 (48%), DENV-4 (30%), DENV-1 (20%), DENV-3 (2%)	First report of DENV-4 in Saudi Arabia; 5.5% of donors were PCR-positive	[16]
Makkah	2017	Cross-sectional	25 suspected patients	Information not specified	24% DENV positive	DENV-1 (50%), DENV-2 (33.3%), DENV-3 (16.6%)	First report of DENV-1 in Makkah; Low prevalence attributed to sample timing/disease stage	[37]
Jazan	2018	Cross-sectional	123	Febrile patients	64.2% DENV positive	DENV-1 (79.7%), DENV-2 (16.5%), concurrent DENV1 + DENV2 (3.8%)	First detection of concurrent mixed infections (DENV1 and DENV2) in Jazan	[38]
Jazan	2018	Cross-sectional	189	Primary infection mostly in <15 years; Secondary infection in 25–65 years	23.3% primary (IgM); 41.8% secondary (IgG); 34.9% negative	DENV-1, DENV-2 (and one concurrent infection)	Secondary infections detected throughout the year; significant increase in April	[39]
Multiregional	2019	Cross-sectional (Community-based)	6596	Older age (>30 years); Residence in Makkah or Jizan; Living in social/popular housing	26.7% IgG positive	Not specified	Seroprevalence highest in Jizan (33.6%), lowest in Madinah (14.8%); Risk factors: absence of pest control, presence of mosquitoes, absence of awareness campaigns	[40]
Najran	2020	Cross-sectional	410 pregnant women	8.5% had a history of travel	7.6% IgG positive; All newborns negative	Not specified	All positive samples were negative by PCR, indicating past infection; Higher prevalence among non-travelers	[41]
Jazan	2021	Cross-sectional	192	Male (56.8%); Age group 21–40 years	RT-PCR positive samples only	DENV-2 (76.6%), DENV-3 (17.2%), DENV-1 (6.3%)	DENV-2 predominant; Regional variation: DENV-1 highest in middle sector, DENV-2 in northern sector, DENV-3 in western sector	[42]
Makkah	2021	Cross-sectional	1004 (2017), 752 (2018), 1571 (2019)	Age 25–44 years; Male predominance; Higher incidence in non-Saudi males in 2019	47.6% positive in 2019 (highest of the 3 years)	Not specified	2019 showed an early and higher peak of cases linked to rainfall and increased *Aedes* density	[18]
Jeddah	2022	Cross-sectional	600	Mostly male (67.5%); Mostly Saudi (71.8%)	22.3% IgG; 10.8% IgM	Not specified	Established a link between gastrointestinal parasites and dengue hemorrhagic fever	[43]
Jazan	2022	Cross-sectional	6637	Age 21–30 years; Male (62.8%)	56.6% positive	Not specified	Highest number of cases in 2019 (44%); 93% of 100 randomly selected suspected cases confirmed by RT-PCR	[44]
Jeddah	2022	Cross-sectional	1458 reported cases	Majority male (77%); Adults 25–44 years; Mostly non-Saudi (64%)	38.1% confirmed cases	Not specified	Spatial clustering in middle and east Jeddah; Temporal peaks in June and December; Environmental factors were not significant (possibly due to COVID-19 restrictions)	[45]
Multiregional	2025	Cross-sectional	2850	Male (73.5%); Adults aged 21–40 years	34% of PCR+ cases were IgG+/PCR+ (secondary infections)	Not specified	Mapped secondary infections; Highest proportion in Asir, Jazan, Makkah; All deaths occurred in IgG+ patients with comorbidities	[14]
Medina	2025	Cross-sectional	202 (117 confirmed)	Male (64.1%); Working-age adults (36–50 years)	57.9% confirmed by PCR/NS1/IgM	Not specified	Seasonal peak in summer (92.3% of cases); PCR had highest sensitivity (91.4%)	[46]
Makkah	2025	Cross-sectional	238 positive samples	Mean age 37.65 years; Mostly Saudi (40.34%), followed by Egyptians; Male (77.73%)	81.5% NS1, 78.6% IgM, 44.5% IgG positive	DENV-2 (80.25%), DENV-1 (12.18%), DENV-3 (1.68%), DENV-4 (0.42%)	Found 11 samples non-reactive to serotypes 1–4, suggesting potential novel serotypes	[18]
Multiregional	2026	Cross-sectional	2850	Male (73.5%); Non-Saudis had higher odds of infection (OR 1.17)	50.8% PCR positive	Not specified	2023 epidemic year; Cases peaked in summer; Regional clustering in Makkah, Najran, and Madinah	[47]

Abbreviations: NS1, non-structural protein 1; IgM, immunoglobulin M; IgG, immunoglobulin G; PCR, polymerase chain reaction; OR, odds ratio. Suspected cases refer to individuals presenting with dengue-like symptoms; confirmed cases are those with laboratory confirmation by PCR, NS1 antigen detection, or serology (IgM/IgG).

**Table 2:** Summary of selection pressure analysis on the DENV *E* gene across three serotypes.

Serotype	Method	Positively Selected Sites	Negatively Selected Sites	Total Sites	Significance Threshold
DENV-1	SLAC	0	172	495	*p* < 0.1
	FEL	0	~275	495	*p* < 0.1
	FUBAR	0	495	495	PP ≥ 0.9
	MEME	2 (episodic: 82, 327)	N/A *	495	*p* < 0.1
DENV-2	SLAC	0	~150	~560	*p* < 0.1
	FEL	22	~25	~560	*p* < 0.1
	FUBAR	0	495	495	PP ≥ 0.9
	MEME	3 (episodic: 202, 404, 493)	N/A	495	*p* < 0.1
DENV-3	SLAC	0	~120	568	*p* < 0.1
	FEL	0	~100	568	*p* < 0.1
	FUBAR	0	568	568	PP ≥ 0.9
	MEME	5 (episodic: 96, 207, 209, 215, 455)	N/A	568	*p* < 0.1

* N/A: Not Applicable.

## Data Availability

The original contributions presented in the study are included in the article, further inquiries can be directed to the corresponding author.

## Supplementary Materials

The following supporting information can be downloaded at https://www.mdpi.com/article/10.3390/ijms27136014/s1.

## Abbreviations

The following abbreviations are used in this manuscript:

**Abbreviation**	**Full Form**
DENV	Dengue Virus
DF	Dengue Fever
DHF	Dengue Hemorrhagic Fever
DSS	Dengue Shock Syndrome
*E* gene/protein	Envelope (protein/gene)
KSA	Kingdom of Saudi Arabia
IgM	Immunoglobulin M
IgG	Immunoglobulin G
NS1	Non-structural protein 1
PCR	Polymerase Chain Reaction
ML	Maximum Likelihood
COS	Cosmopolitan (genotype)
GISAID	Global Initiative on Sharing All Influenza Data
NCBI	National Center for Biotechnology Information
