# Supplementary material for: Epidemiological and Evolutionary Dynamics of Dengue Virus in Saudi Arabia: Insights from Three Decades of Molecular and Serological Surveillance
Source: Int J Mol Sci. 2026 Jul 4;27(13):6014. doi: 10.3390/ijms27136014 (PMC13361391; doi:10.3390/ijms27136014)
Supplement: Supplementary file 1 [file ijms-27-06014-s001.zip › Meta-analysis tables- Tables, S1-S6.pdf]

Table S1. Summary of meta-analysis results for dengue seroprevalence in Saudi Arabia.

Statistic	Value
**Number of Studies**	25
**Total Participants**	32,393
**Total Positive Cases**	13,285
**Pooled Seroprevalence**	40.71% (95% CI: 26.96% – 56.10%)
**I^2 (Heterogeneity)**	98.9% (95% CI: 98.7% – 99.0%)
**τ^2**	2.3906
**τ**	1.5462
**Q Test**	2096.80, df = 24, $p < 0.001$

Table S2. Subgroup analysis by region

Subgroup	Number of Studies (k)	Pooled Seroprevalence (%)	95% CI	I^2 (%)	p-value
**Region**					
Jazan	5	54.77%	[46.20%; 63.06%]	89.9%	
Jeddah	6	40.09%	[30.87%; 50.07%]	96.6%	
Makkah	6	76.16%	[17.51%; 97.96%]	94.6%	
Aseer/Jazan	2	5.95%	[0.12%; 76.42%]	99.2%	<0.0001
Najran	1	7.56%	[5.37%; 10.55%]	—	
Multiregional	3	36.65%	[24.08%; 51.34%]	99.6%	
Al-Madina	2	34.30%	[7.24%; 77.74%]	98.9%	

Between-group difference (random effects model): Q = 122.39, df = 6, $p < 0.0001$

Table S3. Subgroup by assay (Random Effects Model)

Assay	Number of Studies (k)	Pooled Seroprevalence (%)	95% CI
RT-PCR	3	**60.52%**	[54.68%; 66.07%]
PCR/NS1/IgM	1	**57.92%**	[51.00%; 64.54%]
IgM/IgG/PCR	1	**53.11%**	[47.50%; 58.65%]
NS1/IgM	1	**47.74%**	[43.60%; 51.91%]
Serology	1	**47.10%**	[45.41%; 48.80%]
IgM/IgG	1	**41.80%**	[34.98%; 48.95%]
Lab	2	**40.88%**	[28.41%; 54.66%]
Confirmed	1	**38.13%**	[35.67%; 40.66%]
PCR	2	**38.50%**	[16.58%; 66.35%]
PCR/IgG	1	**34.00%**	[32.28%; 35.76%]
IgG	4	**25.33%**	[11.23%; 47.63%]

Assay	Number of Studies (k)	Pooled Seroprevalence (%)	95% CI
IgG/IgM	3	**25.05%**	[14.48%; 39.74%]
IgM	1	**0.83%**	[0.42%; 1.65%]

Between-group difference (random effects model): Q = 383.24, df = 14, p < 0.0001

Table S4. Subgroup by Time Period (Random Effects Model)

Time Period	Number of Studies (k)	Pooled Seroprevalence (%)	95% CI
2003-2010	3	**84.55%**	[14.46%; 99.44%]
2019-2026	11	**45.73%**	[26.76%; 66.02%]
2011-2018	11	**27.11%**	[14.00%; 45.93%]

Between-group difference (random effects model): Q = 3.48, df = 2, p = 0.1760

Table S5. Subgroup by Population (Random Effects Model)

Population	Number of Studies (k)	Pooled Seroprevalence (%)	95% CI
Febrile	17	49.78%	[40.84%; 58.72%]
Healthy	3	13.46%	[0.89%; 72.98%]
Blood donors	2	24.93%	[8.00%; 55.91%]
Community	1	26.70%	[25.64%; 27.78%]
Mixed	1	16.52%	[12.99%; 20.78%]
Pregnant	1	7.56%	[5.37%; 10.55%]

Between-group difference (random effects model): $Q = 110.39$, $df = 5$, $p < 0.0001$

Table S6. Publication Bias (Egger's Test)

Statistic	Value
**t-value**	-0.80
**df**	23
**p-value**	0.4310
**Bias Estimate**	-2.3228 (SE = 2.8978)

Interpretation: The p-value (0.4310) is greater than 0.05, indicating no statistically significant publication bias.
