# Supplementary material for: Epidemiological and Evolutionary Dynamics of Dengue Virus in Saudi Arabia: Insights from Three Decades of Molecular and Serological Surveillance
Source: Int J Mol Sci. 2026 Jul 4;27(13):6014. doi: 10.3390/ijms27136014 (PMC13361391; doi:10.3390/ijms27136014)
Supplement: Supplementary file 1 [file ijms-27-06014-s001.zip › Table S7.pdf]

Table S7. List of DENV-1 Strains used in the phylogenetic analysis

DENV-1			
**Genotype**	**Accession Number**	**Country / City**	**Year**
	FJ810415	Venezuela	2005
	FJ850113	Nicaragua	2005
	JN903579	India	2008
	JX669473	Brazil	2001
	JX669475	Brazil	2002
	KF184975	Angola	2013
	KJ189304	Colombia	2005
	KJ189366	Puerto Rico	2010
	KJ189368	Mexico	2012
	MN244555	Trinidad	2014
	OR145316	Nepal	2022
**America-Africa Genotype**	OR145319	Nepal	2022
	PP626442	Brazil	2022
	PP957577	Colombia	2021
	PQ140588	Peru	2023
	PQ467821	Peru	2024
	PV490568	Mexico	2024
	PV717372	Kenya	2022
	PX297364	India	1970
	PX442212	France	2022
	AM746218	Jeddah	1994
	AM746219	Jeddah	1994
	AM746220	Jeddah	1994
	AB111077	Japan	2002
	AF298808	Djibouti	1998
	GQ398255	Singapore	2008
	JN054255	Sri Lanka	2010
	JN054256	Sri Lanka	2009
**Asian Genotype**	JN415511	Malaysia	2005
	JN697058	Malaysia	2005
	KC759167	China	2012
	KC848578	Somalia	2011
	KF887994	Thailand	2013
	AM746212	Jeddah	2006

DENV-1			
**Genotype**	**Accession Number**	**Country / City**	**Year**
	AM746213	Jeddah	2006
	AM746214	Jeddah	2005
	AM746215	Jeddah	2005
	AM746216	Jeddah	2004
	AM746217	Jeddah	2004
	KJ649286	Jeddah	2011
	KF921948	Viet Nam	2008
	KF955446	Viet Nam	2008
	OR494329	Uruguay	2023
	OZ249546	China	2025
	PP389946	China	2023
	PQ614841	Cambodia	2019
	PV662994	Indonesia	2024
	PX297366	Taiwan	1987
	PX461623	Somalia	2022
	AB074761	Japan	2001
	AB189120	Indonesia	1998
	AB189121	Indonesia	1998
	EF654110	South Korea	2006
	GQ868602	Philippines	2004
	JN415517	Philippines	2010
**South Pacific Genotype**	JN415532	Australia	2009
	JQ915076	French Polynesia-Tahiti	2009
	LC831870	Philippines	2014
	PQ868290	USA	2024
	PV662995	Indonesia	2024
	PX492104	Nauru	1974
	PX683075	Solomon Islands	2025
