# Supplementary material for: Epidemiological and Evolutionary Dynamics of Dengue Virus in Saudi Arabia: Insights from Three Decades of Molecular and Serological Surveillance
Source: Int J Mol Sci. 2026 Jul 4;27(13):6014. doi: 10.3390/ijms27136014 (PMC13361391; doi:10.3390/ijms27136014)
Supplement: Supplementary file 1 [file ijms-27-06014-s001.zip › Table S8.pdf]

Table S8. List of DENV-2 Strains used in the phylogenetic analysis

Genotype	Accession Number	Country / City	Year
	PP732400	Peru	2022
	PQ140595	Peru	2022
	PP732401	Peru	2022
	PQ140594	Peru	2022
	EPI_ISL_2033284	Brazil	2025
	EPI_ISL_2033855	Brazil	2025
	EPI_ISL_2035841	Brazil	2025
	PV451688	Brazil	2024
	PV451689	Brazil	2024
	PQ851446	Colombia	2024
	EPI_ISL_2032076	Colombia	2025
	EPI_ISL_2032077	Colombia	2025
	OR167116	Nepal	2022
	EPI_ISL_2034042	Japan	2025
	PV544691	China	2024
	PQ433671	Taiwan	2023
	PQ433676	Taiwan	2023
	PV544690	China	2024
**COS**	PV663124	Costa Rica	2023
	EPI_ISL_19825443	Jeddah	2021
	EPI_ISL_19825442	Jeddah	2021
	EPI_ISL_19825444	Jeddah	2021
	EPI_ISL_19825445	Jeddah	2021
	EPI_ISL_19825447	Jeddah	2021
	AM746226	Jeddah	1994
	PX297377	Jeddah	1994
	EPI_ISL_19825450	Jeddah	2021
	EPI_ISL_19825449	Jeddah	2021
	EPI_ISL_19825448	Jeddah	2021
	EPI_ISL_19825446	Jeddah	2021
	EPI_ISL_18081191	Saudi Arabia	1994
	OR389324	Saudi Arabia	1994
	EPI_ISL_19825441	Jazan	2023
	PX297377	Jeddah	1994
	AM746225	Jeddah	1994
	AM746227	Jeddah	1994

Genotype	Accession Number	Country / City	Year
	AF410378	Saudi Arabia	1992
	AM746221	Jeddah	2004
	AM746222	Jeddah	2004
	AM746223	Jeddah	1994
	AM746224	Jeddah	1994
	KT175140	Saudi Arabia	2014
	LC416035	Saudi Arabia	2018
	KJ830750	Jeddah	1994
	EPI_ISL_773037	Jeddah	2016
	OR178537	Nepal	2022
	OR167115	Nepal	2022
	EPI_ISL_2034033	Japan	2025
	EPI_ISL_2034037	Japan	2025
	EPI_ISL_2034030	Japan	2025
	EPI_ISL_2034038	Japan	2025
	EPI_ISL_2034043	Japan	2025
	EPI_ISL_2034029	Japan	2025
	EPI_ISL_2034035	Japan	2025
	PX447457	Sri Lanka	2025
	PX447456	Sri Lanka	2025
	PX447461	Sri Lanka	2025
	PX447463	Sri Lanka	2025
	PV554927	India	2024
	PV927173	Indonesia	2024
	EPI_ISL_2034036	Japan	2025
	EPI_ISL_2034026	Japan	2026
	EPI_ISL_2034039	Japan	2025
	OZ285451	Malaysia	2006
	OZ285494	Malaysia	1997
	OZ285502	Malaysia	2002
	PX683041	Solomon Islands	2023
	PX683043	Solomon Islands	2023
	OZ285448	Malaysia	2007
	OZ284272	Viet Nam	2006
	OZ285483	Malaysia	2006
	AY858035	Indonesia	2004
	PX701190	Maldives	2024

Genotype	Accession Number	Country / City	Year
	OZ285442	Malaysia	1989
	OZ285479	Malaysia	1997
	PX480330	Philippines	2019
	PX480307	Philippines	2018
	PX480308	Philippines	2018
	PX824458	Burkina Faso	1986
	L10051	Somalia	1984
	L10044	Indonesia	1976
	PX297373	Indonesia	1988
	PX297370	India	1974
	OZ285443	Malaysia	1989
	PX297374	Malaysia	1983
	PX461635	Somalia	2023
	AF359579	China	1999
	OR178938	Nepal	2010
	PX309104	France	2018
**ASI**	D181805	Thailand	1979
	PV663125	Thailand	2024
	X15434	Malaysia	1987
	D00345	Thailand	1989
	PX297375	Myanmar	1976
	D10514	Thailand	1958
	PQ008452	China	2022
	L10052	Taiwan	1987
	PX297378	Taiwan	1987
**ASII**	L10043	Philippines	1983
	AF038403	New Guinea	1944
	KM204118	New Guinea	1944
**AM/AS**	AF398106	Venezuela	1998
	PX297379	Venezuela	1998
	AF163096	Venezuela	1996
	M20558	Jamaica	1983
	PX297381	Viet Nam	1987
	PQ363536	Colombia	2019
	PQ833551	Colombia	2023
	PQ851445	Colombia	2024
	PP732442	Peru	2016

Genotype	Accession Number	Country / City	Year
	PP732443	Peru	2016
	AF208496	Martinique	1998
	PQ363531	Colombia	2014
	PQ363532	Colombia	2011
	PV628996	Costa Rica	2023
	PV628997	Costa Rica	2023
	GU588123	Honduras	2007
	FJ898461	Belize	2002
	AY449682	Mexico	2001
	GU586492	Guatemala	2007
	DQ364515	El Salvador	1999
	DQ364514	Costa Rica	2000
	GQ199895	Nicaragua	1999
**AM**	L10053	Trinidad	1953
	L10043	India	1957
	PX297369	India	1963
	AY744147	Tonga	1974
	L10046	Puerto Rico	1969
	AF100469	Mexico	1992
	AF100465	Venezuela	1987
	PX297380	Venezuela	1987
**Sylv**	AF231717	Malaysia	1970
	AF231718	Ivory Coast	1980
	AF231719	Guinea	1981
	PV791120	Senegal	1999
	PV791121	Senegal	1999
	PV791122	Senegal	1999
	PV791123	Senegal	1999
