# Supplementary material for: Epidemiological and Evolutionary Dynamics of Dengue Virus in Saudi Arabia: Insights from Three Decades of Molecular and Serological Surveillance
Source: Int J Mol Sci. 2026 Jul 4;27(13):6014. doi: 10.3390/ijms27136014 (PMC13361391; doi:10.3390/ijms27136014)
Supplement: Supplementary file 1 [file ijms-27-06014-s001.zip › Table S9.pdf]

**Table S9.** List of DENV-3 Strains used in sequence and phylogenetic analysis

| Genotype | Accession Number | Country / Location | Year |
|---|---|---|---|
| Genotype 1 | PP897403 | Spain | 2019 |
| | PX297384 | Indonesia | 1986 |
| | PX297389 | Malaysia | 1990 |
| | PQ146648 | Indonesia | 2019 |
| | PQ146647 | Indonesia | 2019 |
| | PQ146634 | Indonesia | 2018 |
| | PQ146633 | Indonesia | 2018 |
| | AY858043 | Indonesia | 2004 |
| | EU081223 | Singapore | 2005 |
| | AY858037 | Indonesia | 2004 |
| | AY858034 | Indonesia | 2004 |
| | MN420515 | Australia | 2008 |
| | KY921906 | Singapore | 2015 |
| | MH823209 | Indonesia | 2018 |
| | MN295851 | China | 2020 |
| | MN292041 | China | 2019 |
| | MF629927 | China | 2016 |
| | MF682968 | China | 2016 |
| Genotype 2 | EU367962 | China | 2007 |
| | OR05976 | Thailand | 2008 |
| | KU508200 | Thailand | 2011 |
| | AY496877 | Bangladesh | 2002 |
| Genotype 3 | PQ467857 | Peru | 2024 |
| | PQ467864 | Peru | 2024 |
| | PQ467863 | Peru | 2024 |
| | PV221118 | Mexico | 2024 |
| | PQ467856 | French Guiana | 2023 |
| | PP582652 | French Guiana | 2023 |
| | PQ348035 | USA | 2024 |
| | PP935894 | USA | 2024 |
| | PQ617127 | USA | 2024 |
| | PP992461 | USA | 2024 |
| | PP935847 | USA | 2025 |
| | PQ617111 | India | 2025 |
| | PQ462099 | USA | 2025 |

| Genotype | Accession Number | Country / Location | Year |
|---|---|---|---|
| | PQ553469 | Cuba | 2022 |
| | PQ553470 | Cuba | 2022 |
| | PQ617113 | USA | 2024 |
| | PP582675 | French Guiana | 2023 |
| | PP582668 | French Guiana | 2023 |
| | PP582677 | French Guiana | 2023 |
| | PP582651 | French Guiana | 2023 |
| | PP582687 | French Guiana | 2023 |
| | PP582686 | French Guiana | 2023 |
| | PP582676 | French Guiana | 2023 |
| | PP582689 | French Guiana | 2023 |
| | PQ553472 | Cuba | 2022 |
| | PP582462 | USA | 2023 |
| | PX398242 | Haiti | 2023 |
| | PX398243 | Haiti | 2023 |
| | PX398244 | Haiti | 2023 |
| | PP922605 | India | 2022 |
| | PP922604 | India | 2021 |
| | PQ143239 | India | 2023 |
| | PQ143240 | India | 2023 |
| | PX909672 | India | 2021 |
| | PX908181 | India | 2021 |
| | PX904863 | India | 2019 |
| | PX909671 | India | 2021 |
| | PX906179 | India | 2021 |
| | MZ857187 | Saudi Arabia | 2016 |
| | EPI_ISL_19825453 | Jazan | 2023 |
| | EPI_ISL_19825454 | Jazan | 2023 |
| | EPI_ISL_19825451 | Jazan | 2023 |
| | EPI_ISL_19825457 | Jazan | 2023 |
| | EPI_ISL_19825458 | Jazan | 2023 |
| | EPI_ISL_19825455 | Jazan | 2023 |
| | EPI_ISL_19825456 | Jazan | 2023 |
| | KF954940 | China | 2013 |
| | KX380842 | Singapore | 2013 |
| | MK894340 | China | 2018 |
| | OR18507 | Nepal | 2022 |

| Genotype | Accession Number | Country / Location | Year |
|---|---|---|---|
| | OR18505 | Nepal | 2024 |
| | PQ922603 | India | 2021 |
| | AM746231 | Jeddah | 2004 |
| | AM746232 | Jeddah | 2004 |
| | AM746233 | Jeddah | 2004 |
| | KF041258 | Pakistan | 2009 |
| | MK051806 | Kenya | 2019 |
| | AM746229 | Jeddah | 2004 |
| | JN662391 | China | 2014 |
| | LC379194 | Gabon | 2016 |
| | PX454667 | Mali | 2023 |
| | PX454660 | Mali | 2023 |
| | PX454665 | Mali | 2023 |
| | PX454658 | Mali | 2023 |
| | PX454657 | Mali | 2023 |
| | PX454679 | Mali | 2023 |
| | PX454678 | Mali | 2024 |
| | MF146273 | Thailand | 2015 |
| | PX445783 | Myanmar | 2018 |
| | PV663135 | Thailand | 2023 |
| | PQ357511 | Thailand | 2023 |
| | PV663138 | Thailand | 2024 |
| | KY588227 | Thailand | 2009 |
| | AM746228 | Jeddah | 1997 |
| | AM746230 | Jeddah | 1997 |
| | PQ682705 | Sri Lanka | 2008 |
| | PX282478 | Saint Kitts-Nevis | 2009 |
| | PP881497 | Colombia | 2024 |
| | PQ983008 | Colombia | 2023 |
| | PQ851406 | Colombia | 2024 |
| | KJ807051 | Jeddah | 2014 |
| **Genotype 5** | PX297385 | Philippines | 1998 |
| | MB3130 | Philippines | 1958 |
| | KU51764 | China | 1980 |
| | KU050695 | Philippines | 1956 |
| | JQ929554 | USA | 1963 |
