# Supplementary figures and images for: Epidemiological and Evolutionary Dynamics of Dengue Virus in Saudi Arabia: Insights from Three Decades of Molecular and Serological Surveillance
Source: Int J Mol Sci. 2026 Jul 4;27(13):6014. doi: 10.3390/ijms27136014 (PMC13361391; doi:10.3390/ijms27136014)

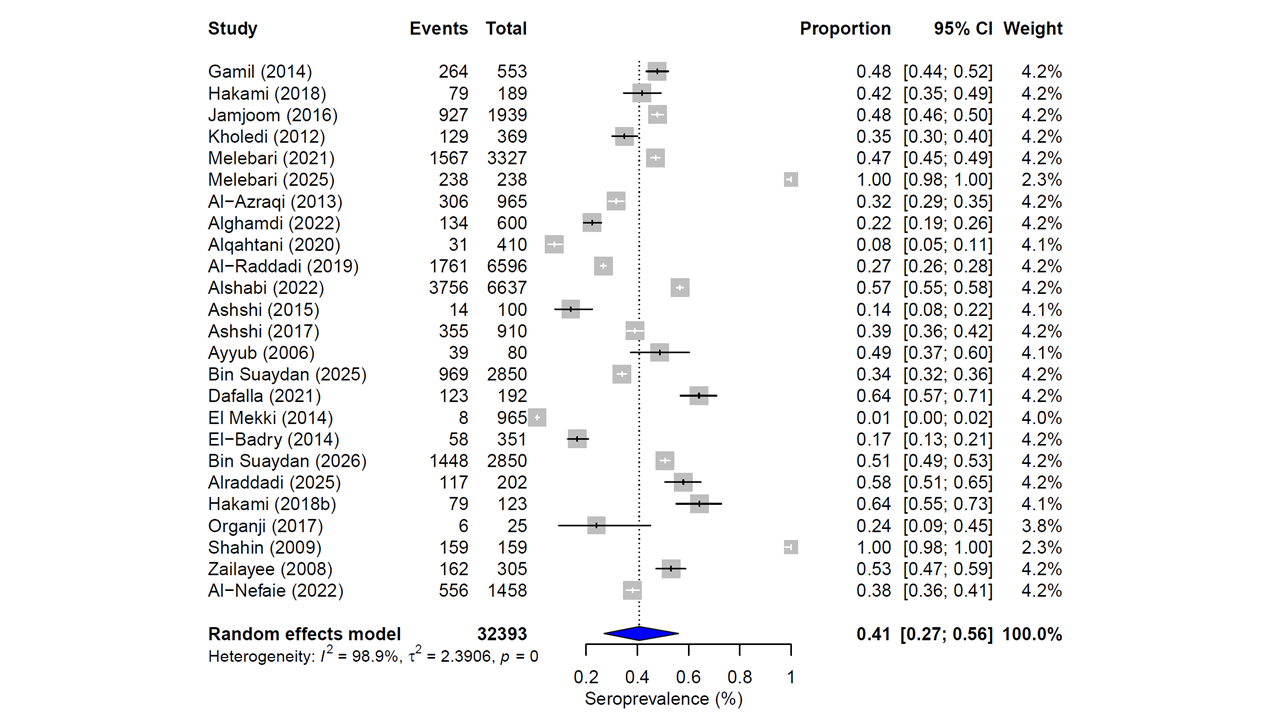

Supplement: Supplementary file 1 [file ijms-27-06014-s001.zip › Figure S1-Forest plot of the pooled seroprevalence of dengue virus infection in Saudi Arabia.TIF]

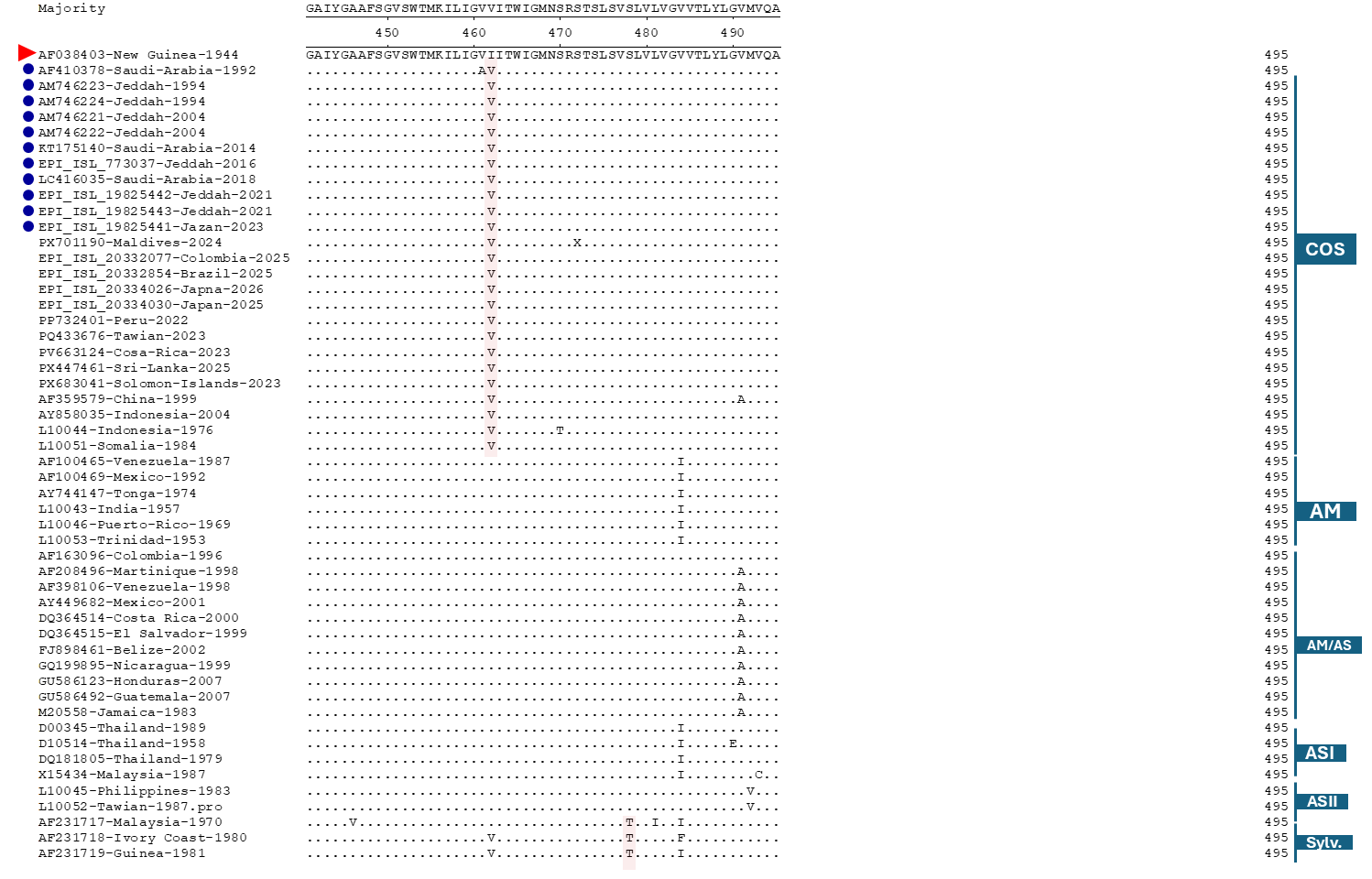

Supplement: Supplementary file 1 [file ijms-27-06014-s001.zip › Figure S10. multiple aligment of DENV-2 amino acids.TIF]

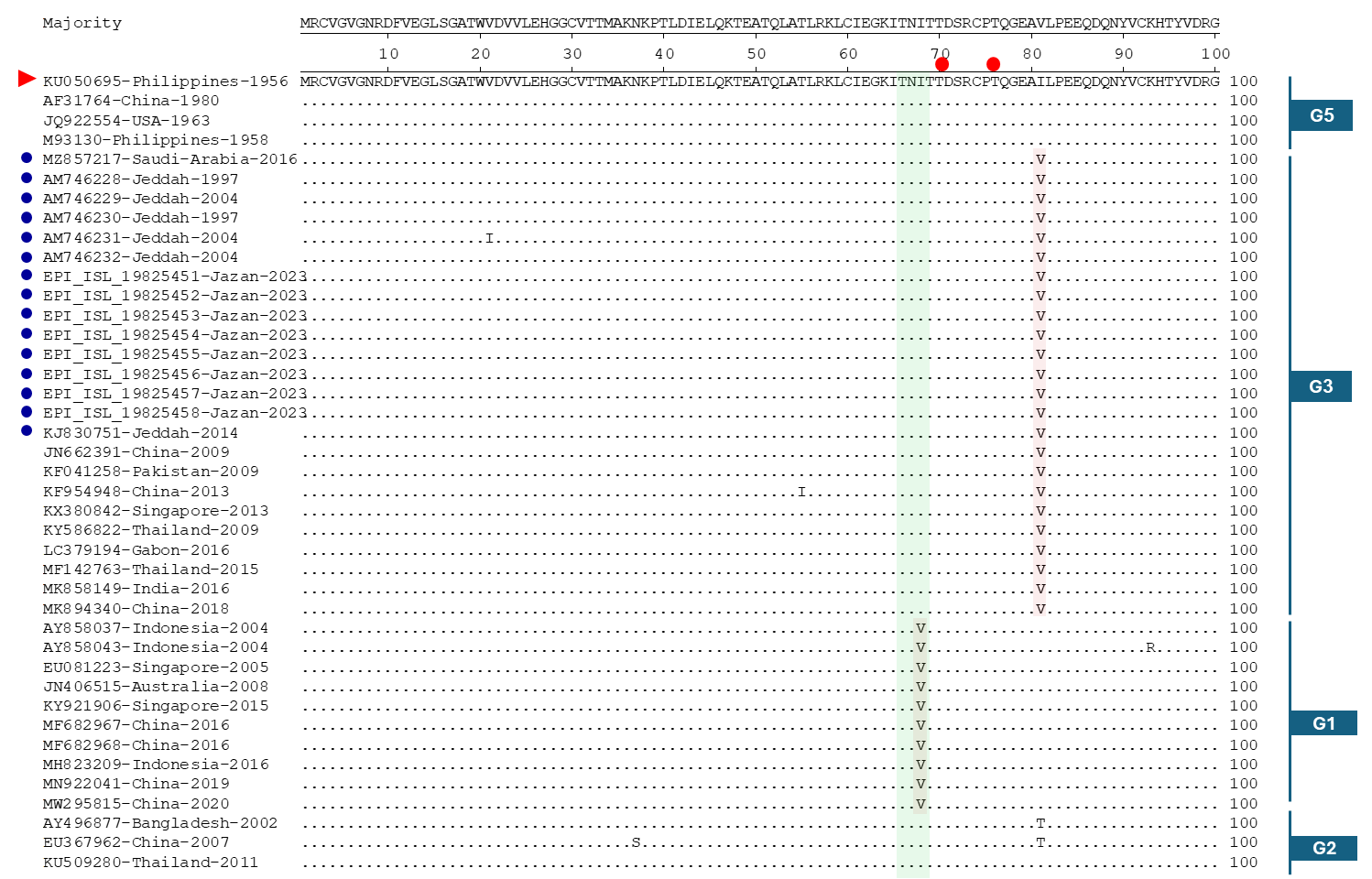

Supplement: Supplementary file 1 [file ijms-27-06014-s001.zip › Figure S11. multiple aligment of DENV-3 amino acids.TIF]

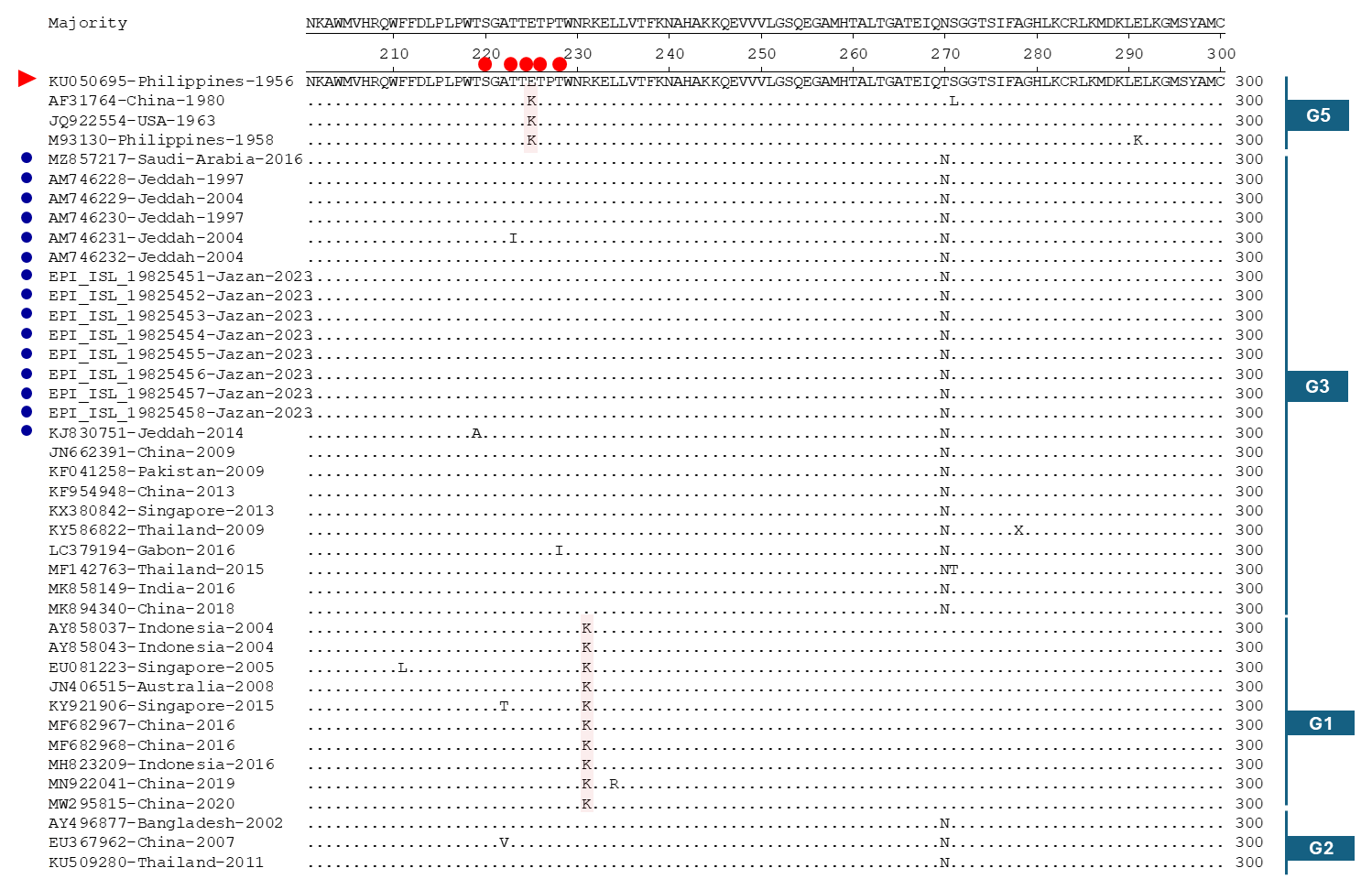

Supplement: Supplementary file 1 [file ijms-27-06014-s001.zip › Figure S12. multiple aligment of DENV-3 amino acids.TIF]

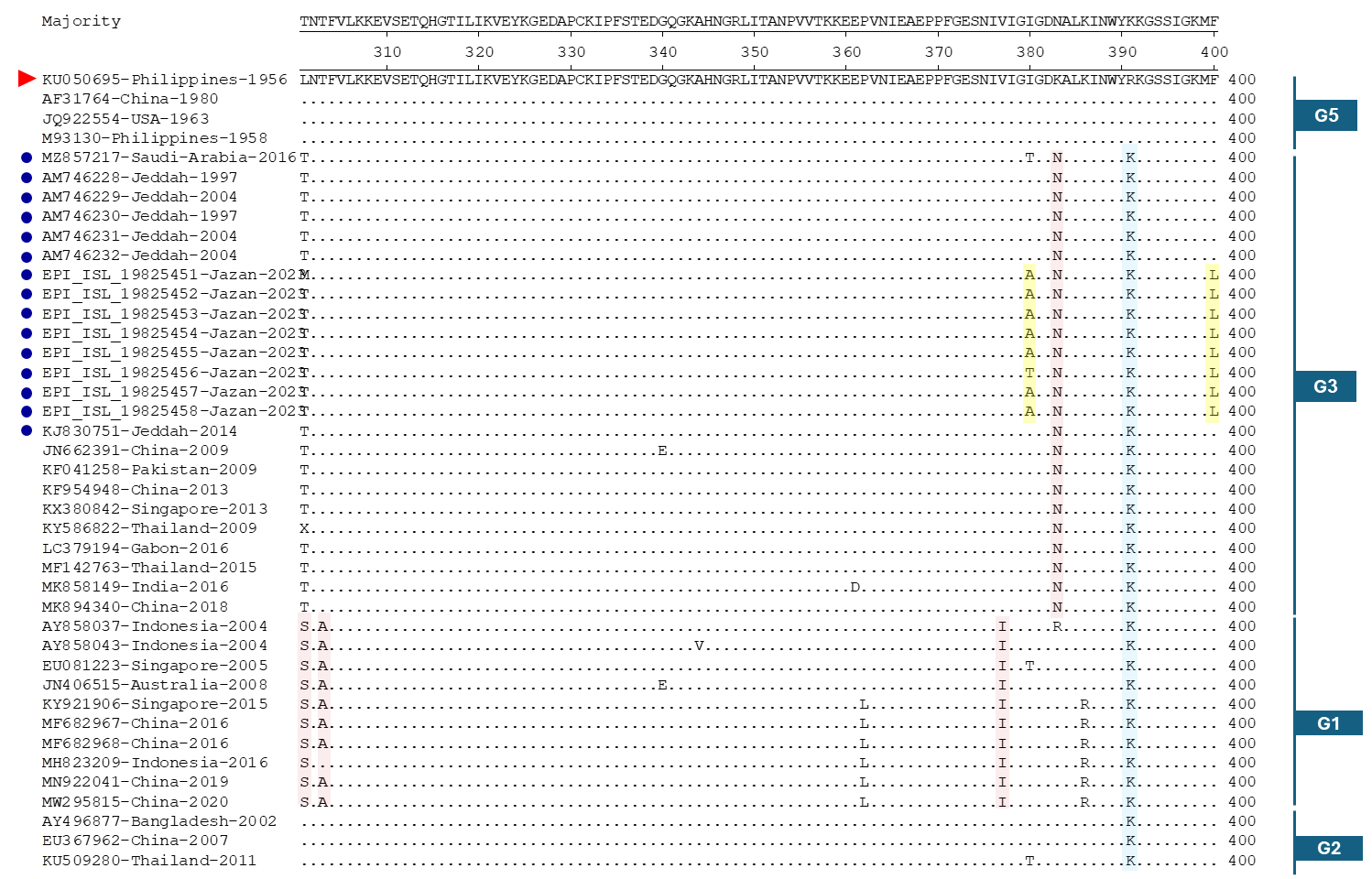

Supplement: Supplementary file 1 [file ijms-27-06014-s001.zip › Figure S13. multiple aligment of DENV-3 amino acids.TIF]

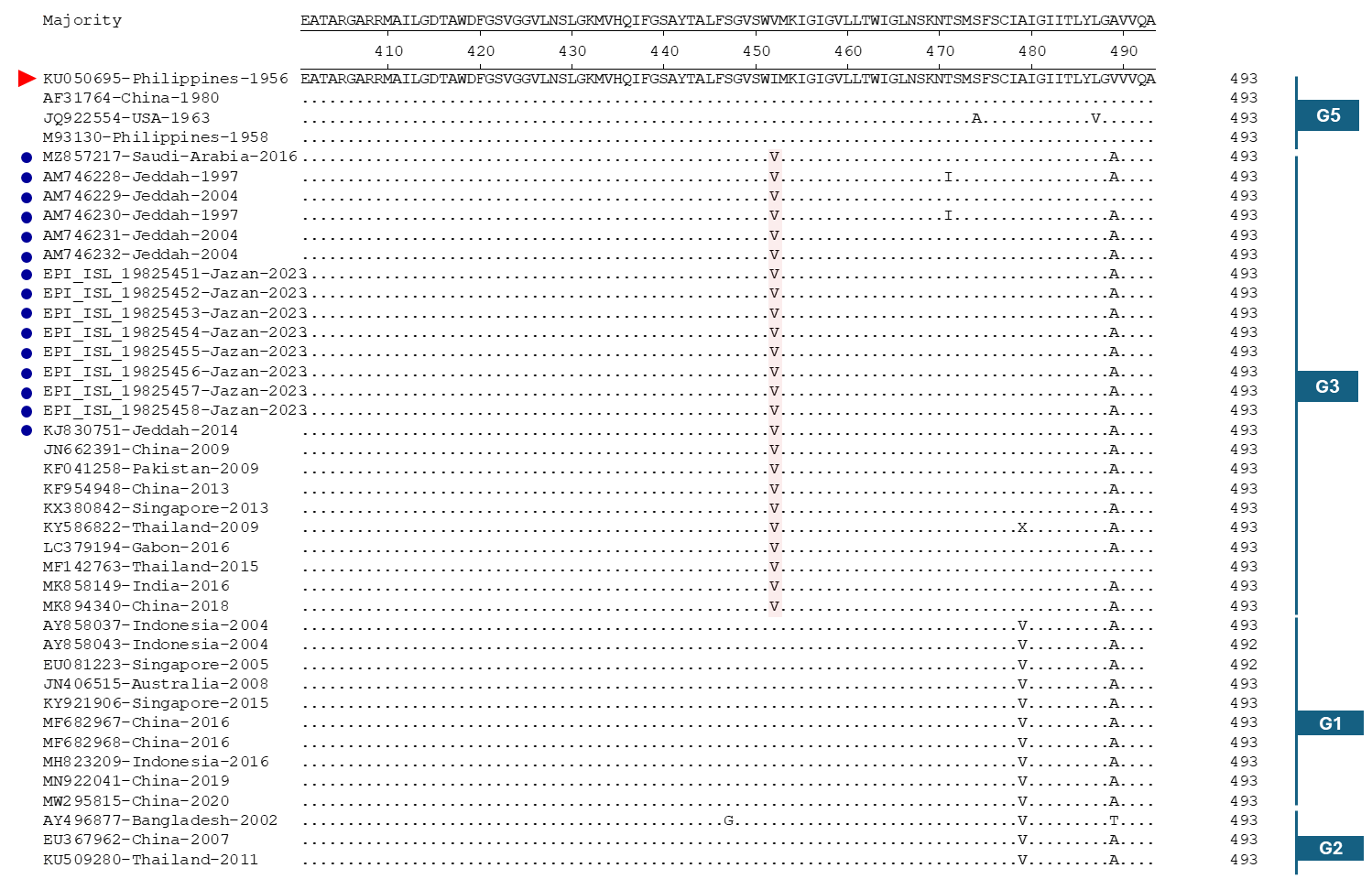

Supplement: Supplementary file 1 [file ijms-27-06014-s001.zip › Figure S14. multiple aligment of DENV-3 amino acids.TIF]

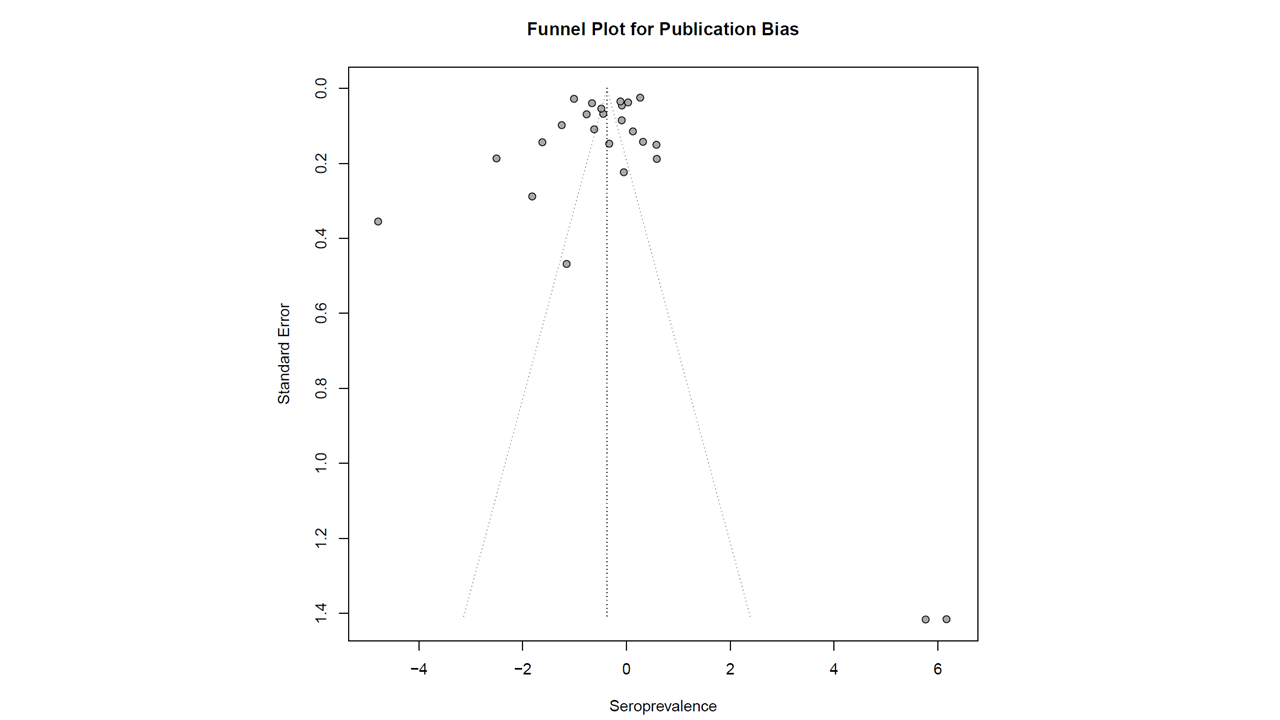

Supplement: Supplementary file 1 [file ijms-27-06014-s001.zip › Figure S2. Funnel plot for publication bias assessment..TIF]

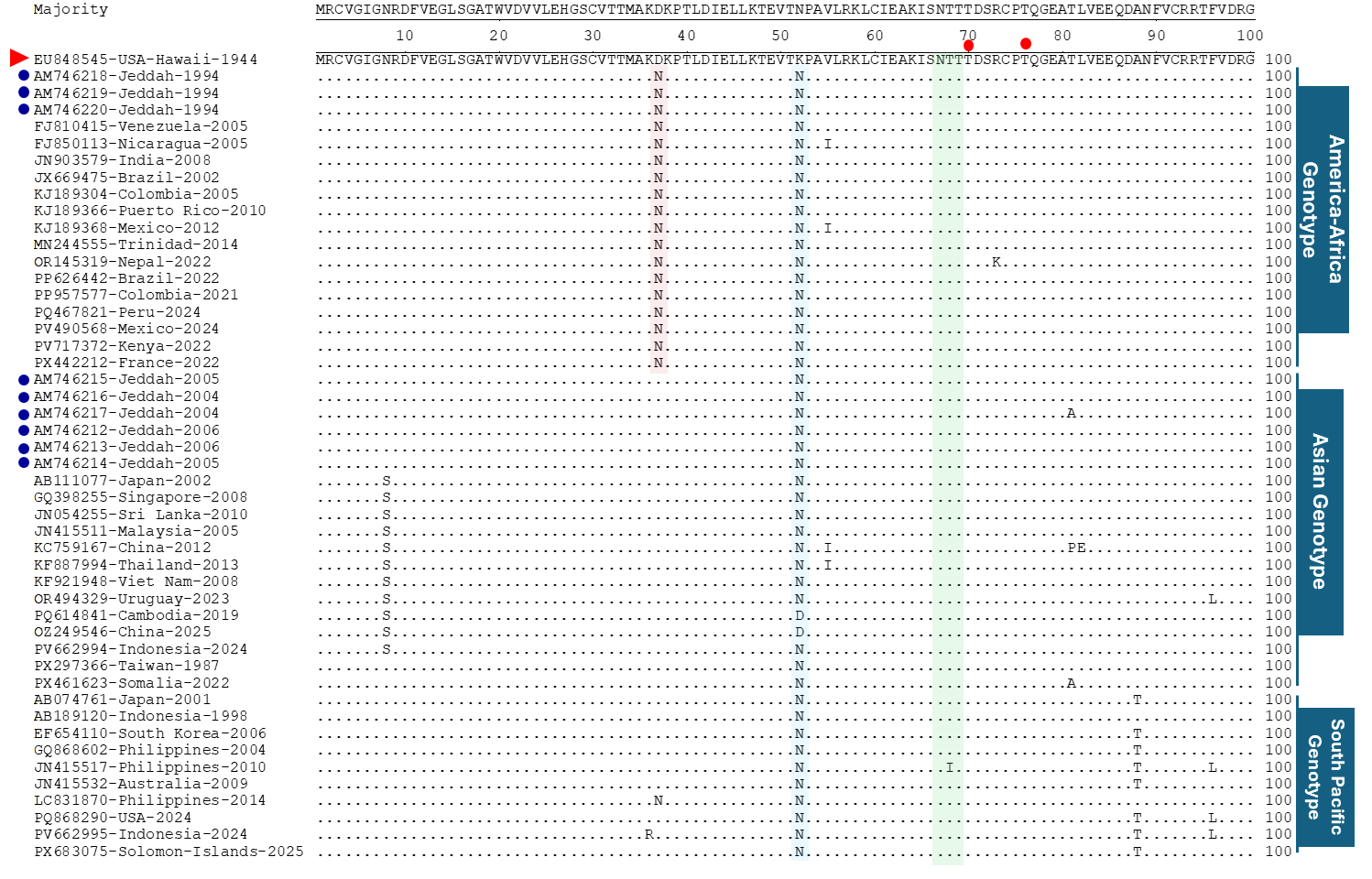

Supplement: Supplementary file 1 [file ijms-27-06014-s001.zip › Figure S3. multiple aligment of DENV-1 amino acids.TIF]

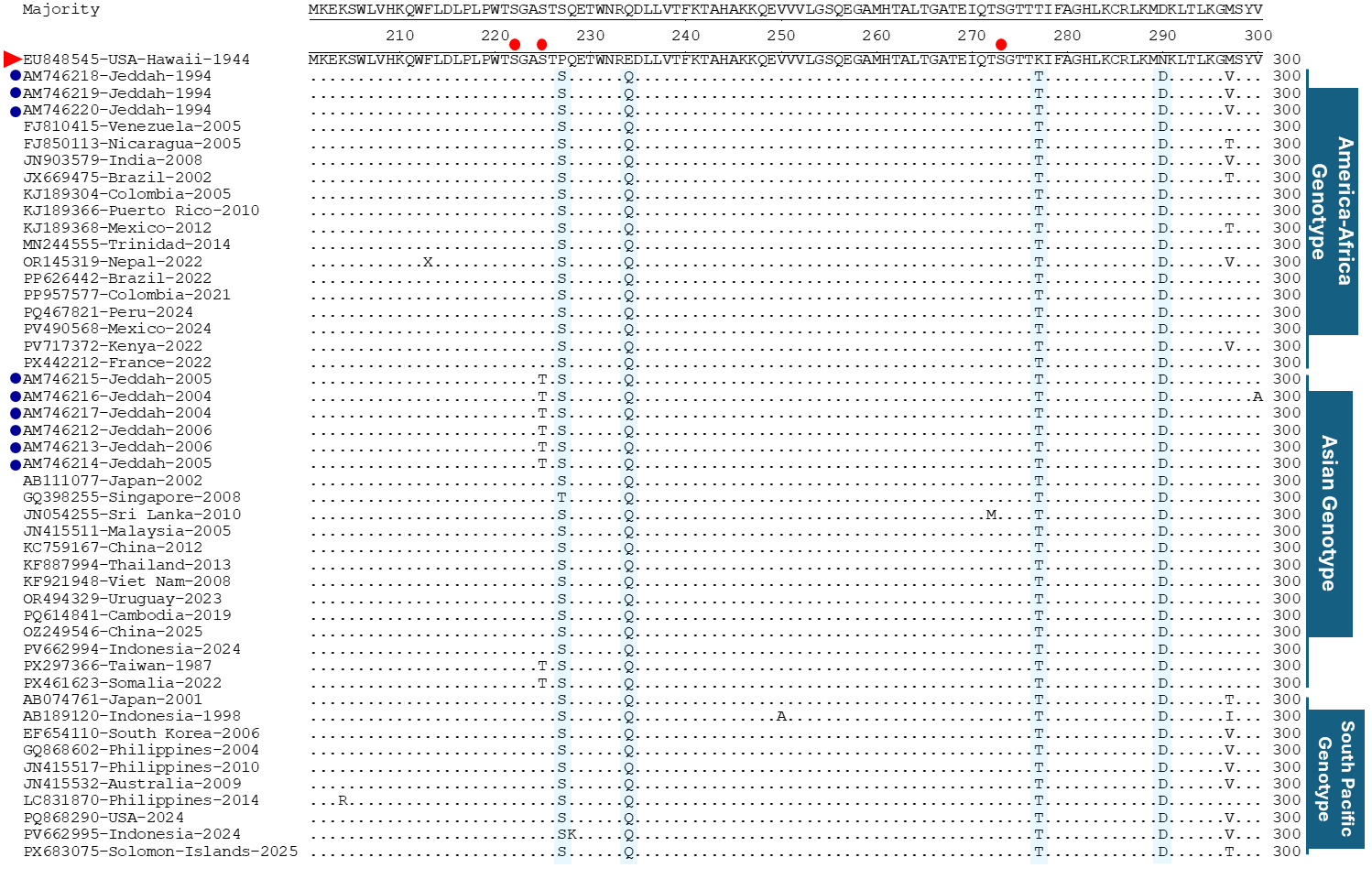

Supplement: Supplementary file 1 [file ijms-27-06014-s001.zip › Figure S4. multiple aligment of DENV-1 amino acids.TIF]

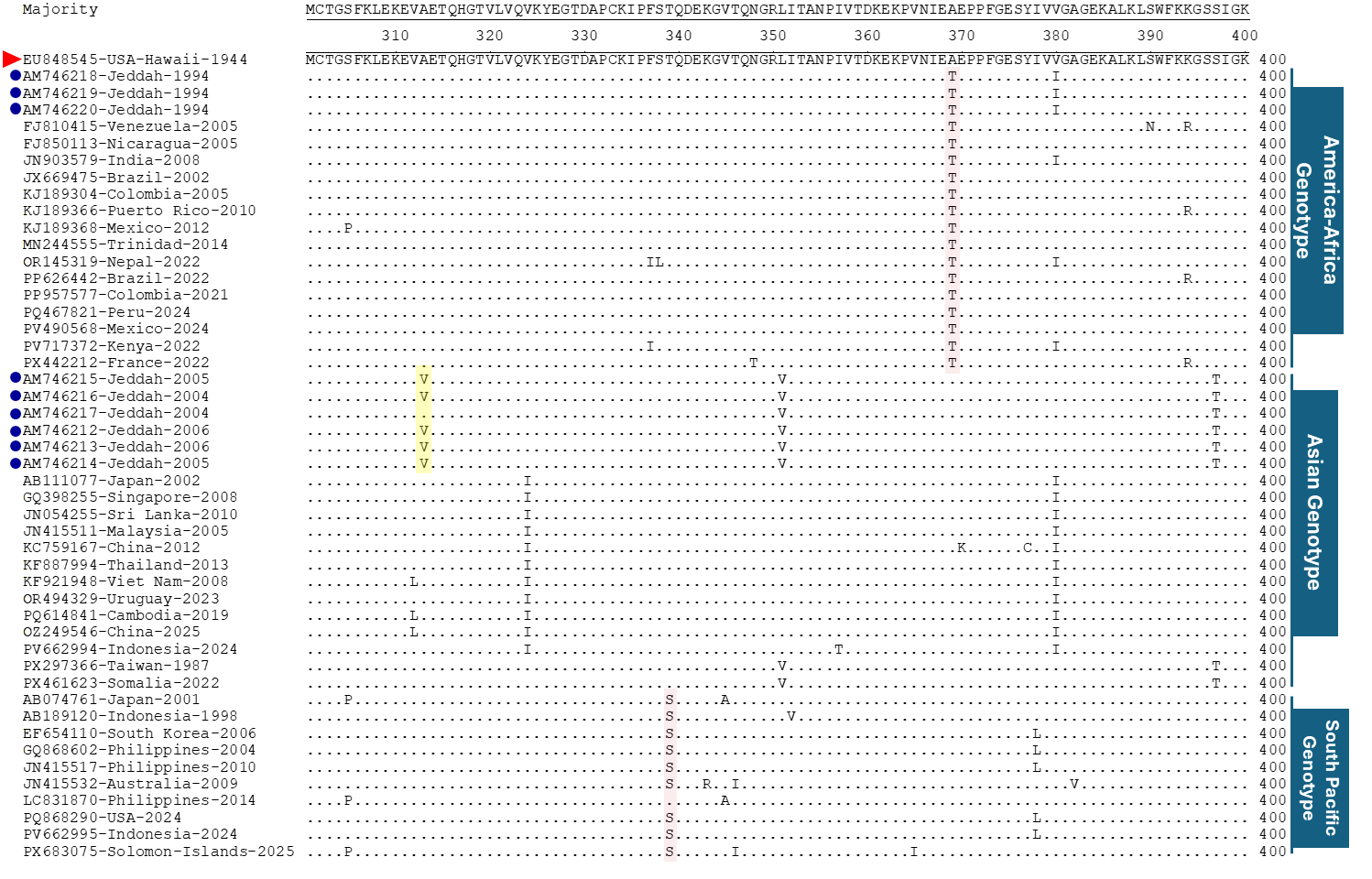

Supplement: Supplementary file 1 [file ijms-27-06014-s001.zip › Figure S5. multiple aligment of DENV-1 amino acids.TIF]

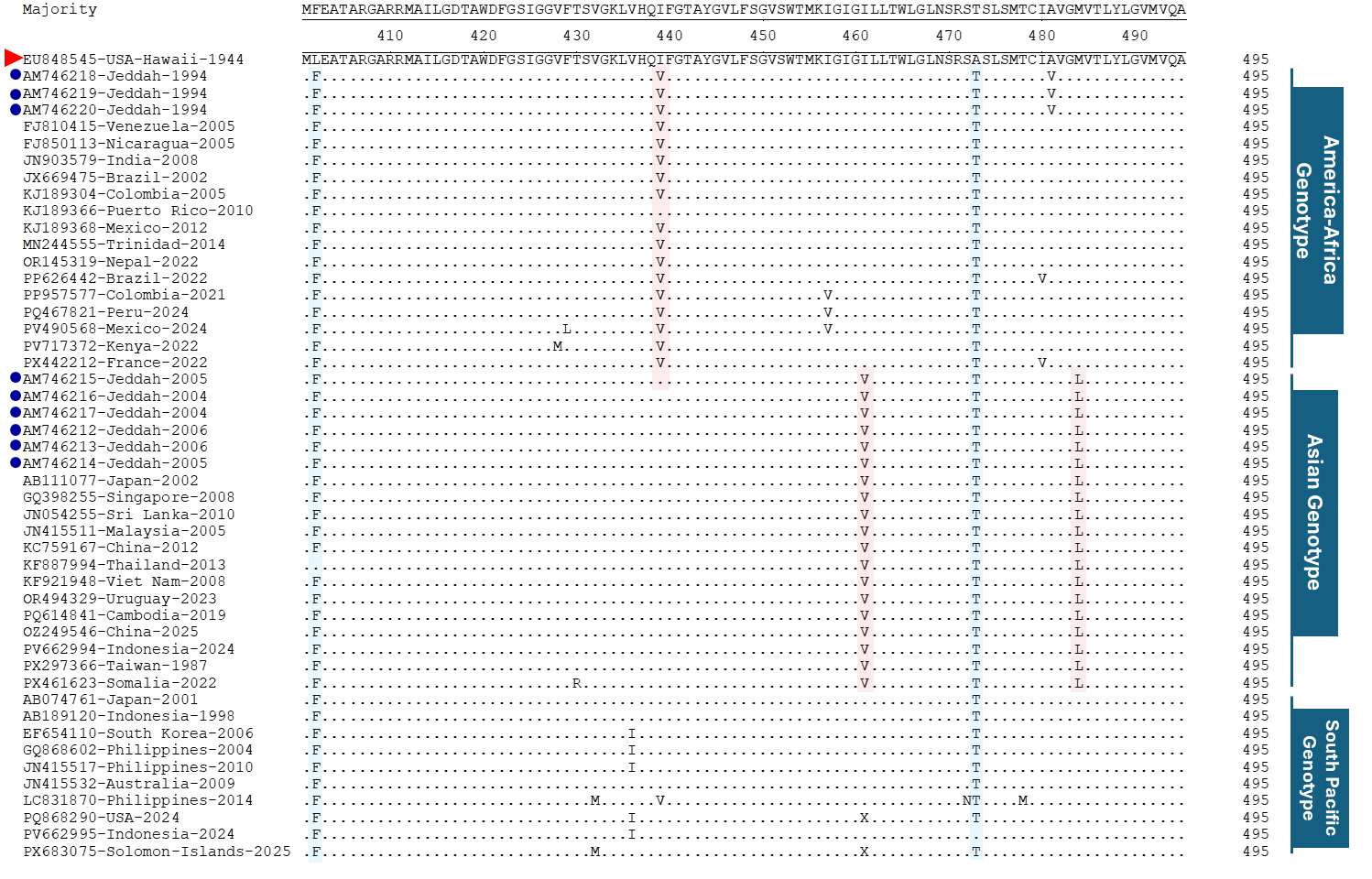

Supplement: Supplementary file 1 [file ijms-27-06014-s001.zip › Figure S6. multiple aligment of DENV-1 amino acids.TIF]

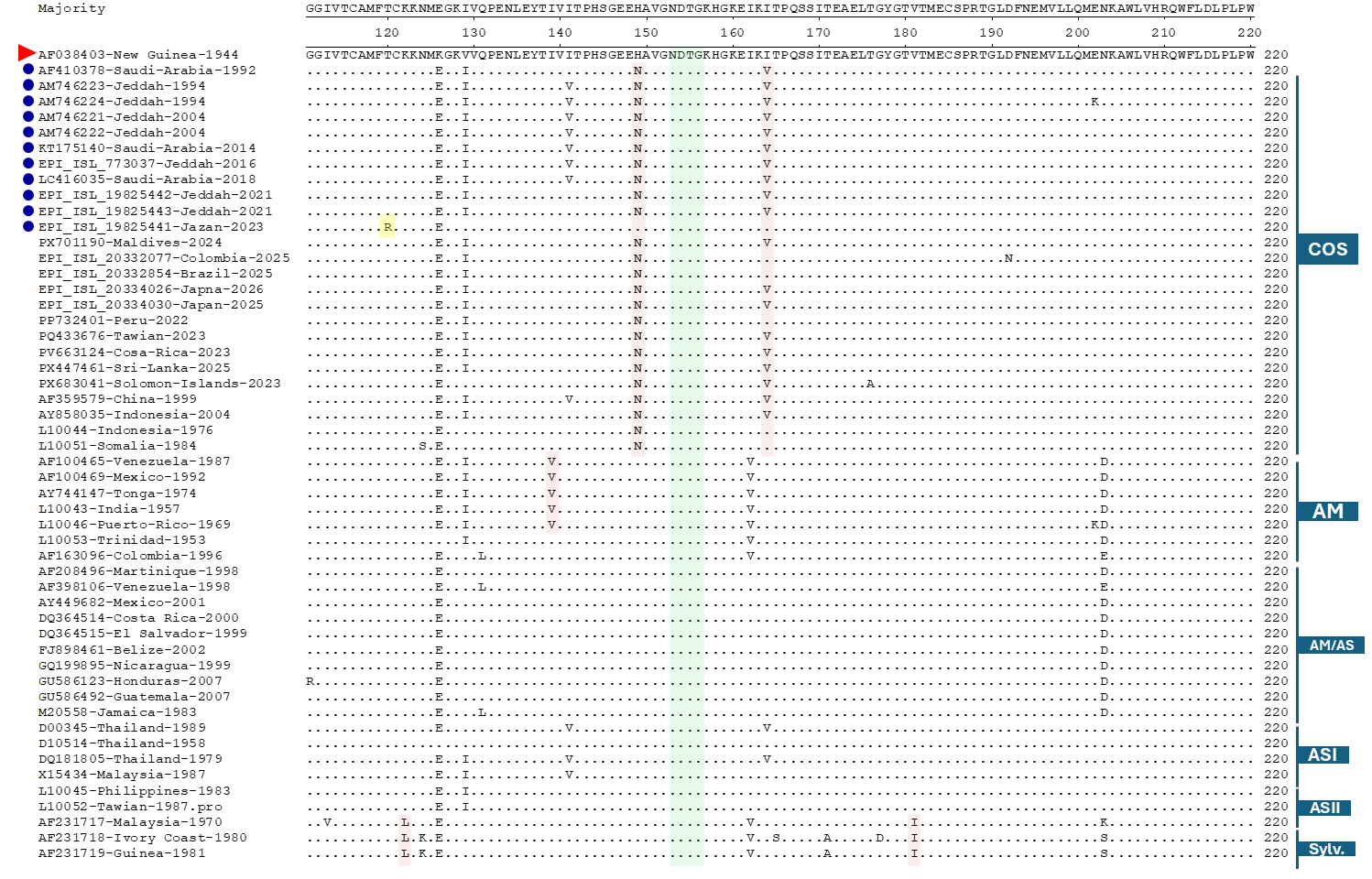

Supplement: Supplementary file 1 [file ijms-27-06014-s001.zip › Figure S7. multiple aligment of DENV-2 amino acids.TIF]

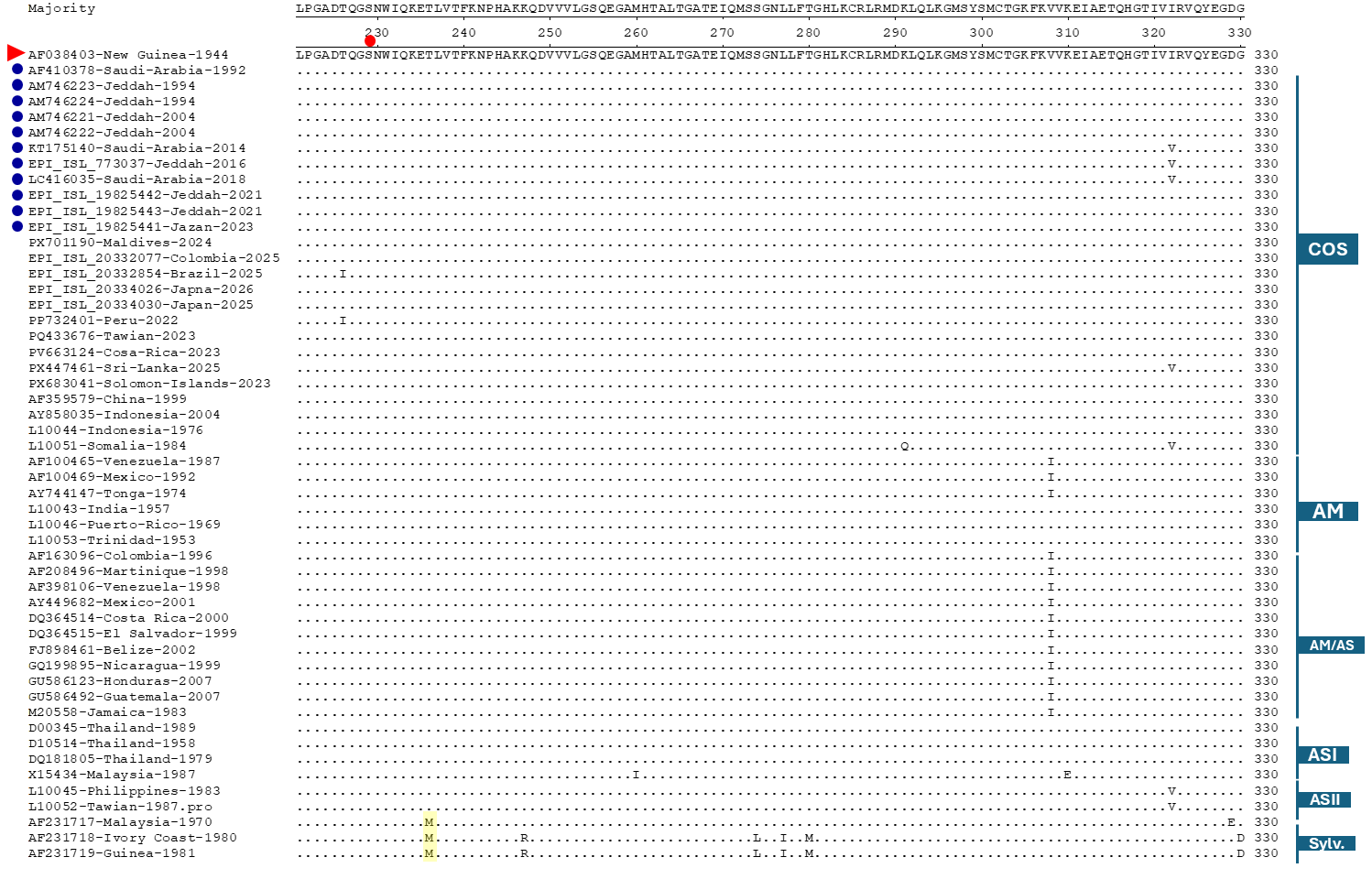

Supplement: Supplementary file 1 [file ijms-27-06014-s001.zip › Figure S8. multiple aligment of DENV-2 amino acids.TIF]

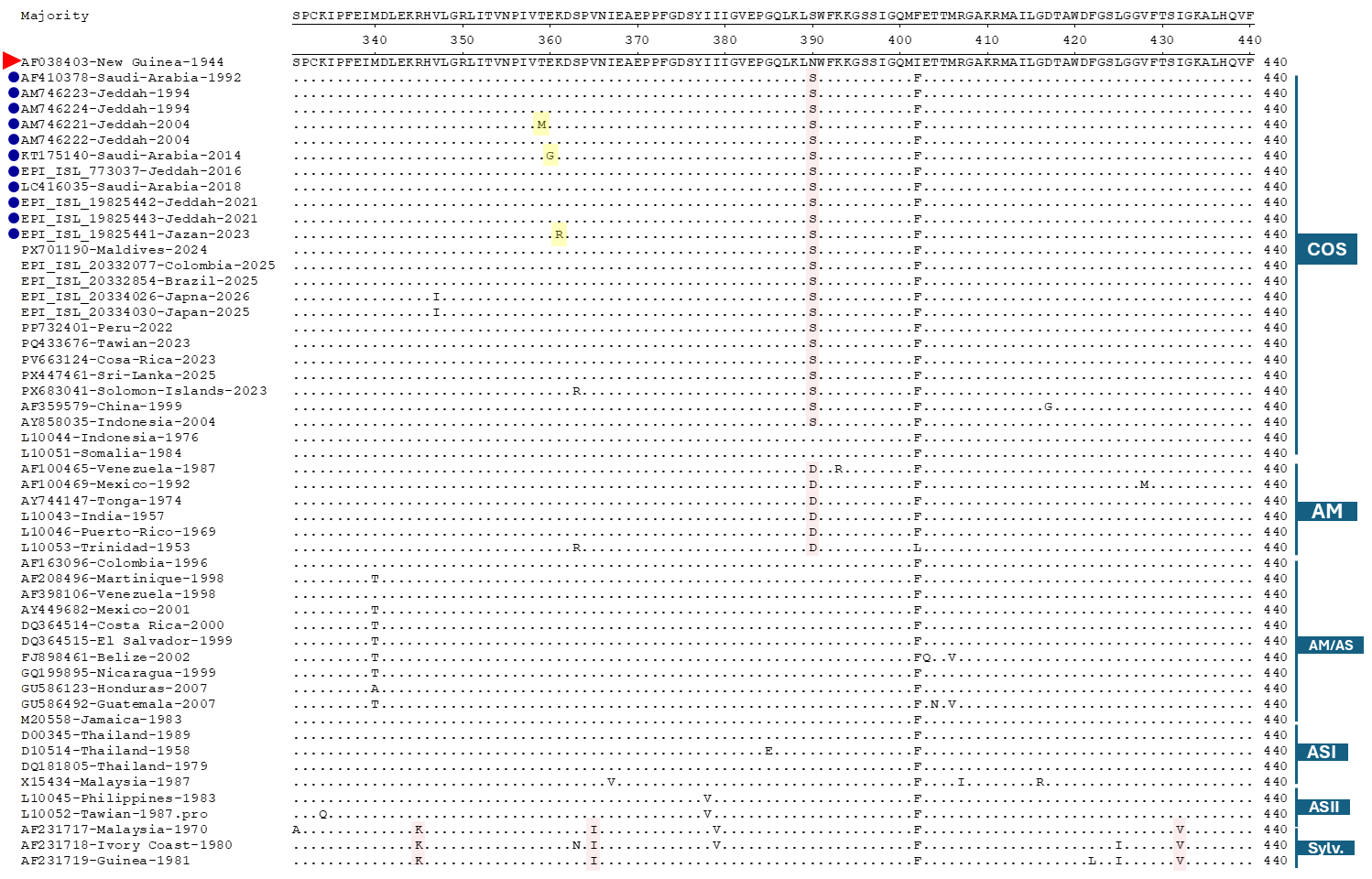

Supplement: Supplementary file 1 [file ijms-27-06014-s001.zip › Figure S9. multiple aligment of DENV-2 amino acids.TIF]
